# FKBP9 Drives Hepatocellular Carcinoma EMT by Regulating SEPT6 Transcription and Stability to Activate TGF-β Signaling

**DOI:** 10.7150/ijbs.134156

**Published:** 2026-09-11

**Authors:** Yu Zhang, Rixin Su, Jie Zou, Wangyang Sun, Banglong Song, Long Liu, Shuanghu Yuan

**Affiliations:** 1 Department of Radiation Oncology, The First Affiliated Hospital of USTC, Division of Life Sciences and Medicine, University of Science and Technology of China, Hefei, Anhui, 230001, China.; 2 Department of Radiation Oncology, Anhui Provincial Cancer Hospital, Hefei, Anhui, 230031, China.; 3 Department of Oncology, The First Affiliated Hospital of Anhui Medical University, Hefei, Anhui, 230001, China.; 4 Department of Surgery, University of Pennsylvania Perelman School of Medicine, 3400 Civic Center Boulevard, Philadelphia, PA 19104, USA.; 5 Department of Hepatobiliary and Pancreatic Surgery, The Second Affiliated Hospital of Zhejiang University School of Medicine, Hangzhou, Zhejiang, 310000, China.

**Keywords:** hepatocellular carcinoma, epithelial-mesenchymal transition, FKBP9, SEPT6, pinyonomycin

## Abstract

Epithelial-mesenchymal transition (EMT) drives hepatocellular carcinoma (HCC) progression, yet key EMT-driving genes specifically acting in malignant epithelial subpopulations remain poorly characterized. Here, we systematically screened EMT-related genes with cancer cell-specific high expression in HCC, and investigated the functional roles and downstream mechanisms of FKBP9. We found that FKBP9 was specifically highly expressed in HCC cancer cell subpopulations, and functionally promoted HCC cell proliferation, clonogenicity, migration and EMT progression. Mechanistically, FKBP9 enhanced SEPT6 transcription by upregulating c-MYC, and directly bound to SEPT6 protein to increase its stability, which in turn activated the TGF-β1 signaling cascade to drive EMT. We further validated the preclinical anti-HCC efficacy of pinyonomycin as a putative FKBP9-targeting inhibitor. Our findings indicate that FKBP9 drives HCC progression and EMT via the SEPT6-mediated TGF-β pathway, highlighting FKBP9 as a promising therapeutic target for HCC.

## Introduction

Liver cancer remains a highly prevalent malignant tumor with dismal prognosis worldwide, with a 5-year overall survival (OS) rate consistently below 30% 1,2. Hepatocellular carcinoma (HCC) accounts for 75-85% of all primary liver cancer cases 3, and its complex biological behavior and heterogeneous molecular mechanisms pose major challenges to clinical management. While therapeutic advances in surgery, chemotherapy, locoregional therapy, immunotherapy, and targeted therapy have expanded treatment options for HCC patients, the long-term clinical benefit remains unsatisfactory for most advanced cases 4. Therefore, identifying key driver genes in HCC pathogenesis and developing targeted therapeutic strategies are critical to improving risk stratification, early intervention, and clinical outcomes for HCC patients.

Epithelial-mesenchymal transition (EMT) has emerged as a pivotal process in tumor progression and metastasis across multiple cancer types 5. During EMT, cancer cells lose epithelial cell-cell tight junctions and acquire mesenchymal migratory and invasive phenotypes, primarily via transcriptional repression of the epithelial marker E-cadherin 6. Beyond driving metastasis, EMT also confers chemo- and radioresistance to tumor cells by inhibiting apoptosis and upregulating ABC transporter expression 7. Despite extensive evidence linking EMT to HCC progression, existing studies have largely focused on bulk tumor tissues, with limited investigation into the cell-type-specific expression and function of EMT-related genes within HCC cancer cell subpopulations. Most of the previously reported EMT-related genes are also widely expressed in immune cells and stromal cells in the tumor microenvironment (TME). Targeting such genes is prone to off-target effects and significant systemic toxicity, which is the key reason for the failure of clinical translation of EMT-targeted therapies. Defining the key EMT-driving genes that act specifically in malignant HCC cells may therefore uncover novel, more precise therapeutic targets with higher translational potential for this devastating disease.

FKBP9 is a member of the FK506-binding protein (FKBP) family, which classically functions in intracellular signal transduction and protein folding as molecular chaperones 8. Previous studies on FKBP9 have mainly focused on glioma, where it promotes tumor metastasis, metabolic reprogramming, and endoplasmic reticulum stress resistance 8-10. While several FKBP family members have been implicated in EMT regulation 11, the functional role and mechanistic involvement of FKBP9 in HCC-associated EMT remain completely unknown. SEPT6, a member of the Septin family with GTP-binding and hydrolysis activity, is involved in cytokinesis, cytoskeletal remodeling, and cell migration 12. While extensively studied in leukemia 13, SEPT6 has been shown to drive epithelial-mesenchymal transition (EMT) via TGF-β pathway activation in multiple solid tumors including lung cancer 14,15. In hepatocellular carcinoma (HCC), only limited studies have reported SEPT6 upregulation and its pro-tumor role via the Hippo/YAP pathway 16, whereas its upstream transcriptional regulation, protein stability control, and involvement in HCC EMT and TGF-β signaling remain largely uncharacterized.

In this study, to fill the critical research gap of FKBP9's functional role in HCC and identify cancer cell-specific EMT drivers for clinical translation, we hypothesized that FKBP9 promotes HCC progression and EMT by regulating SEPT6 to activate the TGF-β signaling pathway. We first systematically analyzed the expression and prognostic value of EMT-related genes in HCC, and identified FKBP9 as a cancer cell-specific EMT-driving gene. We further characterized the prognostic significance and oncogenic function of FKBP9 in HCC *in vitro* and *in vivo*, and dissected its downstream regulatory axis via SEPT6-mediated TGF-β pathway activation. Finally, we evaluated the preclinical therapeutic potential of targeting FKBP9 using its candidate inhibitor pinyonomycin in HCC. Our findings provide novel insights into the cell-type-specific mechanisms of EMT in HCC, and identify FKBP9 as a promising therapeutic target for this devastating disease.

## Methods

### Patients and cells

HCC data from TCGA-LIHC (371 cases) and GSE76427 (167 cases) were collected and combined. Human HCC cell lines Hep3B and Huh7 were obtained from the American Type Culture Collection (ATCC, Manassas, VA, USA) and preserved in GibcoDulbecco modified (DMEM). This study was approved by the Ethics Committee of the Second Hospital of Zhejiang University School of Medicine (Ethics: 2024-0397). This study adheres to the Declaration of Helsinki.

### Cell transfection

Stable knockdown of target genes was achieved by lentiviral short hairpin RNA (shRNA) delivery (Table S1). The pLKO.1 constructs were co-transfected into cells with the packaging plasmids using a calcium phosphate transfection kit (CAPHOS-1KT, Sigma) for shRNA knockdown and overexpression. Viruses were collected, filtered, and titrated before infection of target cells with 8 µg/ml Polybrene (TR-1003, Sigma). The infected cells were screened with puromycin.

### PCR and western blot

Total RNA was extracted from the cells with Trizol Regent (Invitrogen), followed by real-time quantitative PCR in triplicate using the PrimeScript™ RT kit (TakaraBio, Inc.) employing the SYBRPremixExTaq™ kit (TakaraBio) and the ABI7900HTReal-TimePCR system (AppliedBiosystemsLifeTechnologies, FosterCity, CA, USA) for real-time quantitative PCR in triplicate. RT-qPCR primers are shown in Table S2. Homogenization was performed with cell lysis buffer (P0013B, Beyotime, China). The supernatant was collected as protein samples. Protein content was determined using a BCA protein assay kit (P0009, Beyotime, China). Aliquots of protein samples were separated by denaturing 10% SDS-PAGE and transferred to polyvinylidene difluoride (PVDF) membranes. The membrane was then incubated with a specific primary antibody at 4 °C overnight with stirring. The membrane was then incubated with horseradish peroxidase (HRP)-conjugated secondary antibody (1:1000) for 60 min at RT. Finally, protein bands were detected using an enhanced chemiluminescence (ECL) protein blotting detection system (GE healthcare, Amersham, UK). Antibody information is provided in the *Supplementary Material*.

### Co-immunoprecipitation assay

Whole cells were lysed into NP-40 lysis buffer (20 mMTris-HClpH8.0, 137 mMNaCl, 10% glycerol) containing 1% NP-40 and protease inhibitor cocktail (P1005, Beyotime, China) in an ice bath for 10 min. The mixture was sonicated at 30% intensity (10 times for 2 s on ice). The supernatant was collected and incubated with the applicable beads for 2-3 h at 4 °C, then rinsed three times with ice-cold PBS. Finally, samples were boiled in an SDS loading buffer and analyzed by Western blotting.

### Chromatin immunoprecipitation assay

The ChIP was performed according to the ChIP kit (Bes5001, bersinbio, China): Hep3B and Huh7 cells were collected and sonicated to generate DNA fragments of 200 to 500 bp. Lysates were then immunoprecipitated with anti-c-MYC, YY1, or IgG antibodies overnight. The immunoprecipitated DNA was extracted and analyzed by qPCR (Table S3).

### Dosing experiment

Half-life analysis: Cycloheximide (CHX, CAS: 66-81-9, GLPBIO, China) at a final concentration of 100 μg/mL was added to the cell culture medium, and total cellular proteins were collected at 0 h, 6 h, and 12 h after drug addition for western blot quantitative analysis.

For *in vitro* experiments, pinyonomycin (CAS: 55658-47-4, GLPBIO, China) was used at a final working concentration of 1 μg/mL. This concentration was established based on the 72 h half maximal inhibitory concentration (IC50) for cell proliferation in Hep3B and Huh7 cells, which was experimentally determined in the present study. For *in vivo* animal experiments, pinyonomycin was administered via intraperitoneal (i.p.) injection at a dose of 10 mg/kg every other day.

### Animal experiments

All animal experiments in this study were approved by the Institutional Animal Care and Use Committee (IACUC) of Taizhou Hospital Affiliated to Zhejiang University School of Medicine (Ethics: T8Y-2025069). All experimental procedures were performed in strict accordance with the Guide for the Care and Use of Laboratory Animals published by the National Institutes of Health (NIH, USA) and the Animal Research: Reporting of *In Vivo* Experiments (ARRIVE) guidelines. HCC cells in the logarithmic growth phase were harvested to prepare a single-cell suspension. An inoculum of 1 × 10⁶ cells in 100 μL of PBS was injected subcutaneously into the right dorsal flank of nude mice. After anesthesia, a laparotomy was performed on nude mice to expose the left lobe of the liver. An inoculum of 5 × 10⁵ cells in 50 μL of PBS was slowly injected into the subcapsular space of the left hepatic lobe.

### Statistical analysis

Data were presented as mean±standard deviation (SD) of no fewer than three biological replicates. SPSS Statistics 23 and GraphPad Prism 5.0 software were used for statistical analysis and chart generation. Differences between two independent groups were assessed using Student's t-test when normally distributed (determined by D'Agostino-Pearson tests), including Welch correction in case of unequal variances (ascertained using F-test). If not normal, a nonparametric test (Mann-Whitney test) was used. Two-way ANOVA followed by Tukey's post-hoc test was performed to analyze the differences between multiple groups. The survival curves were plotted using the Kaplan-Meier method, and the Cox proportional hazards regression was used to determine the effect of biomarkers on OS and Disease-free survival (DFS), with variance inflation factor analysis. *P*-values < 0.05 were considered statistically significant.

## Results

### FKBP9 is a cancer cell-specific EMT-related gene highly expressed in HCC

To identify the key EMT-related genes specifically expressed in HCC cancer cell subpopulations, we first integrated single-cell RNA sequencing (scRNA-seq) datasets of 3 HCC tissues (GSE107747, GSE223204) and 3 normal liver tissues (GSE223204, GSE174748) (Fig. 1A). After quality control, dimensionality reduction and clustering, we completed cell subpopulation annotation (Fig. S1A), and visualized the distribution and proportion of cell subpopulations across different samples (Fig. S1B). Canonical marker genes were used to accurately distinguish major cell subpopulations, including epithelial cells (cancer cells), immune cells, stromal cells, and endothelial cells (Fig. 1B).

Next, we performed two sets of differential expression analysis to screen cancer cell-specific genes: (1) Differential analysis between HCC cancer cells and normal liver epithelial cells, which generated the cancer cell-specific upregulated gene set DGE-EPC (65 upregulated genes, 196 downregulated genes, Fig. 1C); (2) Differential analysis between HCC cancer cells and non-epithelial cells in the TME, which generated the cancer cell-exclusive high-expression gene set DGE-TME (382 upregulated genes, 703 downregulated genes, Fig. 1D). Meanwhile, we collected 19518 EMT-related genes from the GeneCards database (Fig. 1E). To construct an EMT-related prognostic model and further narrow down the candidate genes, we performed LASSO-Cox regression analysis on the intersecting genes of HCC highly expressed genes and EMT-related genes, and finally included 18 core genes into the prognostic model (Fig. 1F). This EMT model showed excellent stratification ability for HCC patients, with AUC values of 0.853, 0.839 and 0.818 for predicting 1-year, 3-year and 5-year OS, respectively (Fig. S1C-D).

By taking the intersection of DGE-EPC, DGE-TME and EMT-related genes, we obtained a core gene set specifically and highly expressed in HCC cancer cells, including FKBP9 and STC1 (Fig. 1G). Subsequently, we integrated the bulk transcriptome data of TCGA-LIHC (371 cases) and GSE76427 (167 cases), and removed the batch effect using the ComBat method (Fig. 1H). We verified the expression of FKBP9 and STC1 in tumor tissues and paired adjacent non-tumor tissues, and the results showed that both genes were significantly upregulated in HCC tumor tissues (Fig. 1I, Fig. S1E). Kaplan-Meier survival analysis further revealed that high FKBP9 expression, but not STC1, was significantly associated with poor DFS and OS in HCC patients (Fig. 1J-M). Collectively, these results identified FKBP9 as the most prominent candidate that simultaneously met the criteria of "cancer cell-specific expression, core EMT correlation, high expression in bulk tumor tissues, and significant prognostic value".

### FKBP9 enhances the malignant potential of HCC cells

To further verify the effect of FKBP9 on the functions of HCC cells, we performed a series of *in vitro* experiments using two HCC cell lines (Hep3B and Huh7). Firstly, Cell Counting Kit-8 (CCK-8) assay (Fig. 2A) and colony formation assay (Fig. 2B) demonstrated that knockdown of FKBP9 significantly inhibited the proliferation and clonogenic capacity of HCC cells, whereas the opposite effects were observed in cells with FKBP9 overexpression. Subsequently, wound healing assay (Fig. 2C) and Transwell assays (Figs. D-E) confirmed that FKBP9 knockdown remarkably suppressed the migration and invasion abilities of HCC cells, and this inhibitory effect was reversed when FKBP9 was overexpressed. Meanwhile, cytoskeleton staining (Fig. 2F) further revealed that FKBP9 knockdown led to a significant reduction in the expression level of F-actin, accompanied by a rounded change in cell morphology. In contrast, FKBP9 overexpression resulted in increased F-actin expression and a shift in cell morphology to a spindle-like shape. This observation suggests that FKBP9 may promote the epithelial-mesenchymal transition (EMT) of HCC cells. Finally, *in vivo* experiments verified that FKBP9 knockdown inhibited the proliferation and tumor volume of subcutaneous xenografts in nude mice (Fig. 2G). Collectively, these results demonstrate that FKBP9 can promote the proliferation, migration, invasion, and tumorigenic ability of HCC cells, thereby facilitating the malignant progression of hepatocellular carcinoma.

### FKBP9 promotes SEPT6 expression via c-MYC

To further explore the mechanism by which FKBP9 regulates the functions of HCC cells, we performed transcriptome sequencing on HCC cells transfected with FKBP9 shRNA (Sh-FKBP9) and negative control shRNA (Sh-NC) (Fig. 3A). Sequencing results revealed that the differentially expressed genes (DEGs) of FKBP9 were significantly enriched in extracellular matrix (ECM) remodeling and transforming growth factor-β (TGF-β) pathway activation (Figs.3B-C). Among these FKBP9-related DEGs, the expression level of SEPT6 was significantly decreased (ranked 5th, LogFC = -2.601, P = 0.005). SEPT6 is a GTP-binding protein that participates in cytoskeletal organization, cell division, maintenance of cell polarity, and regulation of EMT 14. Additionally, SEPT6 is also highly expressed in epithelial cell populations (Fig. S2A) and has been reported to activate the TGF-β pathway, thereby promoting the EMT process and tumor progression 15. Therefore, we hypothesized that FKBP9 exerts its pro-tumor effects by upregulating SEPT6 expression.

Subsequently, we analyzed the correlation between FKBP9 and SEPT6 expression in HCC using TCGA and GEO datasets. The results showed that SEPT6 expression was significantly higher in the FKBP9 high-expression group, and there was a significant positive correlation between FKBP9 and SEPT6 expression (R = 0.54, P < 0.001) (Figs.3D-E). Single-cell analysis demonstrated highly consistent co-localization of FKBP9 and SEPT6 within epithelial subpopulations (Figs.S2B-D). Meanwhile, PCR verification confirmed that the expression level of SEPT6 in HCC cells exhibited a consistent change with FKBP9 (Fig. 3F). However, the change in SEPT6 protein level was more pronounced compared to its mRNA level (Fig. 3G, Fig. S3A).

To further clarify the regulatory pattern and mechanism of FKBP9 on SEPT6, we first predicted the transcription factors of SEPT6 using three databases (GeneCards, TRAP, and JASPAR). The results suggested that FKBP9 might promote SEPT6 expression through c-MYC and YY1 (Fig. 3H). We then analyzed the effect of FKBP9 on c-MYC and YY1. The results showed that FKBP9 knockdown led to a significant decrease in c-MYC expression, while YY1 expression remained unchanged (Figs.3I-J, Fig. S3B). Therefore, we performed the ChIP assay (Fig. 3K) and Dual-luciferase assay (Fig. S4A) to further investigate the binding of c-MYC to SEPT6. The results demonstrated that c-MYC could directly bind to the SEPT6 promoter.

To further verify that c-MYC is an essential mediator for FKBP9-mediated transcriptional regulation of SEPT6, we performed three sets of rescue experiments. qPCR and Western blotting results showed that FKBP9 overexpression markedly upregulated the mRNA and protein levels of SEPT6, while simultaneous c-MYC knockdown reversed the high SEPT6 expression induced by FKBP9 overexpression (Fig. 3L, Fig. S3C, Fig. S4B). These results definitively validated that c-MYC exerts an irreplaceable mediating role in the regulatory axis of FKBP9-promoted SEPT6 transcription.

Finally, we analyzed the expression profiles of FKBP9, c-MYC and SEPT6 in HCC using the TCGA-LIHC and GSE76427 datasets. The results showed that all three genes were significantly upregulated in tumor tissues (Fig. 1I, Fig. S4C). Correlation analysis revealed a significant positive correlation among FKBP9, c-MYC and SEPT6 in HCC tissues (Fig. 3M), which was further confirmed in the epithelial subpopulation from scRNA-seq data (Fig. S4D). Prognostic analysis indicated that all three genes were significantly associated with the clinical prognosis of HCC patients (Fig. 3N-O, Fig. S4E-F). Taken together, these results demonstrate that FKBP9 promotes SEPT6 transcription via c-MYC, thereby affecting the prognosis of HCC.

### FKBP9 directly binds to SEPT6 and enhances its protein stability

Given that genes of the FKBP family function as molecular chaperones, which are capable of promoting protein folding and stabilization 17-18, and considering the inconsistent changes between SEPT6 protein and mRNA levels following FKBP9 knockdown, we further performed co-immunoprecipitation combined with mass spectrometry (Co-IP/MS) analysis. The results indicated that SEPT6 is one of the potential binding partners of FKBP9 (Fig. 4A). Subsequently, fluorescence co-localization analysis revealed that SEPT6 and FKBP9 exhibit similar expression localizations in HCC cells (Fig. 4B). After transfecting exogenous Flag-FKBP9 and His-SEPT6 plasmids into HEK293T, Hep3B, and Huh7 cells, Co-IP experiments confirmed that exogenous FKBP9 can directly bind to SEPT6 (Figs.4C-D). We further verified the binding between endogenous FKBP9 and SEPT6 in Hep3B and Huh7 cells; the results showed that both endogenous and exogenous FKBP9 can directly bind to SEPT6 (Fig. 4E).

Meanwhile, the DMfold deep learning model predicted that the TYR283, PHE305, and ASP306 residues of FKBP9 can interact with the PRO257, TRP258, and TYR256 residues of SEPT6 (with a pLDDT score of 0.79) (Fig. 4F). To further explore the binding domains between FKBP9 and SEPT6, we divided FKBP9 into three truncation mutants based on its structural features: PPIase (1-150), TPR (151-300), and EF-hand (301-570), and constructed the corresponding plasmids (Fig. 4G). Similarly, SEPT6 was divided into three truncation mutants: GTP (1-150), CC (151-300), and CTD (301-427), with the corresponding plasmids constructed. Co-IP results demonstrated that the TPR domain of FKBP9 and the CC domain of SEPT6 are the essential domains required for their interaction (Figs.4H-I).

To investigate whether FKBP9 regulates SEPT6 via the proteasome pathway, we treated FKBP9-deficient cells with the proteasome inhibitor MG132. Western blotting analysis showed that MG132 treatment markedly restored the reduced SEPT6 protein level caused by FKBP9 deficiency (Fig. S5A). The CHX chase half-life assay confirmed that FKBP9 enhanced the protein stability of SEPT6 (Fig. 4J, Fig. S3D). Furthermore, *in vivo* ubiquitination assay revealed that FKBP9 knockdown significantly increased K48-linked polyubiquitination of SEPT6, while FKBP9 overexpression exerted the opposite effect (Fig. 4K, Fig. S3E). Taken together, these results elucidate the regulatory relationship and mechanism of FKBP9 on SEPT6: FKBP9 not only promotes SEPT6 transcription via c-MYC, but also directly binds to SEPT6 to enhance its protein stability.

### FKBP9 activates the TGF-β pathway via SEPT6

To further verify that SEPT6 is the essential downstream mediator of FKBP9-induced malignant phenotypes and TGF-β pathway activation in HCC cells, we performed a series of rescue experiments with three experimental groups: negative control (Vector+Sh-NC), FKBP9 overexpression alone (FKBP9+Sh-NC), and FKBP9 overexpression combined with SEPT6 knockdown (FKBP9+Sh-SEPT6). We first detected the concentration of TGF-β1 in the supernatant of HCC cells in each group using enzyme-linked immunosorbent assay (ELISA). The results showed that FKBP9 overexpression significantly increased the secretion of TGF-β1 in HCC cells, while this promoting effect was completely abolished by simultaneous knockdown of SEPT6 (Fig. 5A).

To further confirm that FKBP9 activates the TGF-β pathway in a SEPT6-dependent manner, we detected the total protein and phosphorylation levels of Smad2 and Smad3 (core downstream effectors of the canonical TGF-β pathway), as well as EMT-related markers in each group. Western blot results showed that FKBP9 overexpression significantly upregulated the phosphorylation levels of Smad2/3 (without affecting the total protein expression of Smad2/3), downregulated the epithelial marker E-Cadherin, and upregulated the mesenchymal marker N-Cadherin in HCC cells. Notably, all these molecular changes induced by FKBP9 overexpression were markedly reversed by SEPT6 knockdown (Fig. 5B, Fig. S3F). Meanwhile, immunofluorescence staining results revealed that FKBP9 overexpression significantly promoted the nuclear translocation of p-Smad2/3 in HCC cells, and this effect was completely blocked by SEPT6 silencing (Fig. 5C). To further explore the in-depth regulatory effect of the FKBP9-SEPT6 axis on the TGF-β signaling pathway, we detected the TGF-β-induced non-canonical signaling pathways. The results showed that FKBP9 overexpression significantly upregulated the protein levels of p-ERK1/2, p-JNK, p-p38 and p-Akt, while SEPT6 knockdown reversed this effect (Fig. S5B-C). These results indicated that FKBP9 activates the TGF-β signaling pathway and drives EMT progression in HCC cells strictly in a SEPT6-dependent manner.

Subsequently, we validated that SEPT6 is required for FKBP9-induced malignant biological behaviors of HCC cells through *in vitro* functional rescue experiments. CCK-8 assay (Fig. 5D) and colony formation assay (Fig. 5E) showed that FKBP9 overexpression significantly enhanced the proliferation and clonogenic capacity of HCC cells, while these promoting effects were markedly reversed by simultaneous knockdown of SEPT6. Wound healing assay (Fig. 5F-G) and Transwell migration assay (Fig. 5H) confirmed that FKBP9 overexpression dramatically accelerated the migratory ability of HCC cells, and this pro-migratory effect was abolished by SEPT6 knockdown. Similarly, Transwell invasion assay demonstrated that the enhanced invasive capacity induced by FKBP9 overexpression was completely blocked by SEPT6 silencing (Fig. 5I). Furthermore, cytoskeleton F-actin staining showed that FKBP9 overexpression led to a significant increase in F-actin expression, accompanied by a spindle-like mesenchymal morphological change in HCC cells. In contrast, simultaneous knockdown of SEPT6 restored F-actin expression and reversed the mesenchymal morphological transition induced by FKBP9 overexpression (Fig. 5J).

Finally, we validated the regulatory relationship between FKBP9 and SEPT6 *in vivo* using a subcutaneous xenograft tumor model in nude mice. The results showed that FKBP9 overexpression significantly enhanced the tumorigenic capacity of HCC cells, as evidenced by increased tumor volume and weight, while this pro-tumor effect was markedly reversed by SEPT6 knockdown (Fig. 5K). In addition, single-cell cell-cell communication analysis revealed that the FKBP9-high HCC subcluster formed strong and specific crosstalk with fibroblasts and monocytes via paracrine TGF-β signaling (Fig. S6A). Ligand-receptor analysis showed that TGFB1 may drive ECM remodeling and immunosuppression via ACVR1/TGFBR1/TGFBR2 (Fig. S6B). Collectively, these findings demonstrate that FKBP9 promotes the proliferation, migration, invasion, EMT process and tumorigenesis of HCC cells by activating the TGF-β pathway in a SEPT6-dependent manner, confirming that SEPT6 is an essential and indispensable downstream mediator of FKBP9 in HCC.

### Clinical validation of FKBP9 and SEPT6

To further verify the expression correlation and clinical significance of FKBP9 and SEPT6 in clinical tissue samples, we enrolled tumor tissues and paired adjacent non-tumor liver tissues from 97 HCC patients (Table S4) for multiplex immunofluorescence staining analysis. The results showed that the protein expression levels of both FKBP9 and SEPT6 were significantly upregulated in HCC tissues compared with paired adjacent normal liver tissues (Fig. 6A). Meanwhile, an obvious spatial co-localization of FKBP9 and SEPT6 was observed in HCC tissues, while the co-localization level of the two proteins was extremely low in adjacent non-tumor tissues (Fig. 6B). Pearson correlation analysis further confirmed that the protein expression levels of FKBP9 and SEPT6 in HCC tissues were significantly positively correlated (R=0.71, P<0.001, Fig. 6C), which was consistent with the regulatory relationship between the two in HCC cell lines described above.

Subsequently, we divided the 97 HCC patients into high-expression group and low-expression group according to the median protein expression level of FKBP9 and SEPT6 respectively, to analyze the association between their expression and malignant clinicopathological features of HCC. The results showed that patients with high expression of FKBP9 and SEPT6 had significant differences in key malignant clinicopathological phenotypes including tumor diameter, microvascular invasion (MVI), portal vein tumor thrombus (PVT), intrahepatic metastasis, TNM stage, Barcelona Clinic Liver Cancer (BCLC) stage and serum AFP level, compared with the low-expression group (Fig. 6D-E, Table S5-S6), suggesting that high expression of FKBP9 and SEPT6 is closely related to the malignant progression of HCC.

Further prognostic analysis showed that, survival curves plotted by Kaplan-Meier method with log-rank test, combined with univariate Cox regression analysis, revealed that FKBP9 expression, SEPT6 expression, MVI, TNM stage, BCLC stage, tumor differentiation grade, and serum AFP level were all significantly associated with OS of HCC patients, with potential prognostic evaluation value (Fig. 6F, Table S7). Finally, we included the above statistically significant indicators from univariate analysis into multivariate Cox regression analysis (BCLC and SEPT6 were excluded due to high collinearity, Table S8). It was confirmed that high FKBP9 expression (HR = 2.13, 95% CI: 1.24-4.12, P = 0.011) and advanced TNM stage (HR = 4.14, 95% CI: 2.43-6.03, P < 0.001) were independent risk factors for poor OS of HCC patients. Meanwhile, high FKBP9 expression was also an independent risk factor for poor DFS of HCC patients (HR = 2.95, 95% CI: 1.72-5.42, P = 0.004, Fig. 6G, Table S9).

### Pinyonomycin can inhibit the malignant potential of HCC by targeting FKBP9

To further analyze the effectiveness and feasibility of FKBP9 as a target for HCC prevention and treatment, we identified pinyonomycin as a candidate FKBP9-targeting agent using the DSigDB database, and calculated the binding sites of pinyonomycin and FKBP9: TYR285, TYR341, PHE305, and ASP296 in FKBP9 protein by deep learning (Fig. 7A). Subsequently, we further verified the direct binding between pinyonomycin and FKBP9 via isothermal titration calorimetry (ITC) and cellular thermal shift assay (CETSA). ITC assays revealed that pinyonomycin exhibited a distinct binding affinity to the recombinant FKBP9 protein, with a dissociation constant (Kd) of 5.23 ± 0.42 μM (Fig. 7B, Fig. S3G). CETSA assays demonstrated that pinyonomycin markedly enhanced the thermal stability of FKBP9 protein (Fig. 7C, Fig. S3H). Collectively, these findings solidly confirmed that pinyonomycin acts as a direct binding ligand for FKBP9 protein.

Pinyonomycin (Pingyangmycin, bleomycin A5) is a clinically used antitumor antibiotic of the bleomycin class, whose classical mechanism involves inducing DNA single- and double-strand breaks, inhibiting DNA synthesis, and exerting broad cytotoxic effects on rapidly dividing cells 19. However, whether pinyonomycin exerts additional antitumor effects through targeting specific oncogenic proteins in HCC, particularly the FKBP9-mediated EMT pathway, remains completely unknown. We then verified the regulatory effect of pinyonomycin on the FKBP9-SEPT6 axis by PCR and western blot (Fig. 7D-E). Treatment with 1 μg/mL pinyonomycin significantly downregulated the protein expression levels of FKBP9 and SEPT6, and inhibited the phosphorylation of Smad2/3 (a marker of TGF-β pathway activation). Notably, forced overexpression of FKBP9 partially alleviated the pinyonomycin-induced downregulation of SEPT6 and inhibition of TGF-β signaling. These results indicate that pinyonomycin exerts its inhibitory effect on the SEPT6-TGF-β axis, at least in part, through targeting FKBP9. Finally, the effects of the pinyonomycin on HCC cell proliferation (Fig. 7F-G), migration (Fig. 7H-J), and invasion (Fig. 7K) were also analyzed. The results suggested that the addition of pinyonomycin significantly inhibited the proliferation, migration, and invasion of HCC, accompanied by a decrease in F-actin expression and altered roundness (Fig. 7L-M), which was alleviated when FKBP9 overexpression.

Additionally, we validated the anti-tumor efficacy of pinyonomycin in hepatocellular carcinoma patient-derived organoids (HCC-PDOs), a clinically relevant model that faithfully recapitulates human tumor heterogeneity. Treatment with 1 μg/mL pinyonomycin markedly restrained HCC-PDO growth, resulting in significantly reduced organoid volume and compromised structural integrity relative to the control group (Fig. S7A-B).

To further validate the *in vivo* therapeutic efficacy of pinyonomycin, we established two HCC animal models: subcutaneous xenograft tumors and orthotopic liver xenograft tumors, with only two groups set for each model: negative control (NC, solvent-treated) and pinyonomycin-treated group. For the subcutaneous xenograft model (n = 5 per group), pinyonomycin treatment significantly inhibited tumor volume growth and reduced final tumor weight compared with the NC group (Fig. 7N), demonstrating a potent anti-proliferative effect of pinyonomycin on HCC *in vivo*. For the orthotopic liver xenograft model (n = 6), which more closely mimics the *in vivo* microenvironment of HCC, pinyonomycin treatment also markedly suppressed the growth of orthotopic liver tumors (Fig. 7O), validating the anti-tumor effect of pinyonomycin in a more physiologically relevant HCC model. Concurrently, the pinyonomycin group exhibited lower Ki-67 expression levels.

Taken together, these *in vitro* and *in vivo* findings demonstrate that in addition to its classical DNA-damaging cytotoxic effect, pinyonomycin can target FKBP9 to suppress the FKBP9-SEPT6-TGF-β1 signaling axis, thereby contributing to its inhibitory effects on HCC cell proliferation, migration, invasion, EMT progression, and *in vivo* tumor growth. These results provide direct preclinical evidence for FKBP9 as a promising therapeutic target for HCC and lay a foundation for the clinical application of pinyonomycin in HCC treatment.

## Discussion

In this study, we addressed the critical gap of lacking cancer cell-specific EMT drivers for HCC. We first explored the prognostic value of an EMT-related model in HCC, and identified FKBP9 as a key EMT gene specifically highly expressed in HCC cancer cell subpopulations. We then analyzed the effects of FKBP9 on HCC cell proliferation, metabolism and EMT, and revealed its underlying mechanism: FKBP9 drives EMT by promoting SEPT6 transcription via c-MYC and enhancing SEPT6 protein stability through direct binding, which further activates the TGF-β pathway. Finally, we identified pinyonomycin as a FKBP9-targeting inhibitor and verified its preclinical anti-HCC efficacy. These results reveal the function and mechanism of FKBP9 in HCC, deepen the understanding of HCC EMT, and provide a novel therapeutic target for HCC treatment.

The mechanism and value of EMT in HCC development and metastasis have been widely demonstrated 20-21. The use of EMT-related genes to construct prognostic models of HCC has also been attempted and validated 22. Although the clinical significance of EMT in HCC has been well established, the specific EMT-related genes that drive this process in the cancer cell subpopulation of HCC and their underlying mechanisms remain incompletely elucidated. Previous studies were mostly limited to macroscopic HCC tissues without in-depth analyses of subpopulation expression differences of genes, ignoring the complex composition and functional differences of tumor tissues. In this study, in addition to constructing an EMT prognostic model of HCC, the expression of genes in cancer cell subpopulations was precisely localized. The key EMT gene FKBP9 was clearly identified in the cancer cells subpopulation of HCC, which provides a new perspective for exploring and understanding the mechanism of EMT in HCC.

Several members of the FKBP family have been shown to be closely related to EMT. For example, FKBP51 was shown to be involved in EMT and metastatic processes in melanoma through activation of ABCG2^23^. FKBP5 was shown to be involved in EMT and fibrosis in lupus nephritis through activation of SMAD 24. FKBP9, a member of the FKBPs family, was shown to be associated with progression and prognosis significantly of glioma 25, bladder cancer 26, and prostate cancer 27. However, there is a lack of studies on the role and mechanism of FKBP9 in the EMT of HCC. Meanwhile, the FKBP family has been proven to act as molecular chaperones that directly bind to proteins to enhance protein stability. Studies have indicated that FKBP51 and FKBP52 can directly bind to steroid hormone receptors, thereby promoting the stability and activity of these receptors 28-29. In this study, we identified FKBP9 as a key epithelial-mesenchymal transition (EMT)-related gene that is highly expressed in a subset of hepatocellular carcinoma (HCC) cells. Through a series of experiments, we demonstrated that FKBP9 activates the EMT process. Additionally, we clarified the direct binding between FKBP9 and its downstream gene product SEPT6, mapped the binding region, and elucidated the effect of this binding on SEPT6 protein stability. These findings not only uncover additional functions of FKBP9 but also confirm its critical role in the pathogenesis of HCC, suggesting that FKBP9 may serve as a promising therapeutic target.

SEPT6 was shown to be significantly associated with EMT in lung cance and liver cancer 30. Meanwhile, overexpression and stabilization of SEPT6 can significantly promote tumor metastasis and progression 31-33. In this study, we not only demonstrated the role of SEPT6 in activating the TGF-β pathway and EMT of HCC but also revealed the regulatory mode of FKBP9-SEPT6. FKBP9 not only promotes the transcription of SEPT6 by up-regulating c-MYC but also promotes the protein stabilization of SEPT6 by binding directly to it. Meanwhile, FKBP9-SEPT6 also had a cross-regulatory relationship in the proliferation, migration, and EMT of HCC cells. Knockdown of SEPT6 significantly reversed the malignant phenotype and TGF-β pathway activation induced by FKBP9 overexpression. Specifically, considering the physiological role of Septins as the 'fourth component' of the cytoskeleton, SEPT6 may facilitate the exocytosis of TGF-β1 by orchestrating F-actin-dependent vesicle trafficking 28. Furthermore, stabilized SEPT6 might also promote the synthesis of TGF-β1 at the transcriptional level through downstream signaling cascades, thereby forming a positive feedback loop to sustain the activation of the TGF-β signaling pathway. These results reveal a previously unrecognized interaction between FKBP9 and SEPT6, providing a comprehensive mechanistic explanation for the oncogenic role of FKBP9 in HCC.

In conclusion, our study identified pinyonomycin as a potential targeted inhibitor of FKBP9. While its classical anti-tumor mechanism involves DNA damage, our rescue experiments first confirm that pinyonomycin exerts specific anti-HCC effects by suppressing the FKBP9/SEPT6 axis. However, several limitations must be acknowledged. This study relies on a single-center retrospective cohort and preclinical models, which may not fully capture human tumor heterogeneity; thus, multi-center prospective trials and patient-derived organoids (PDOs) are needed. Furthermore, systematic safety evaluations and off-target validations remain pending. Future translational research should focus on Phase I/II trials to assess pinyonomycin's pharmacokinetics and explore combination strategies with immune checkpoint inhibitors (e.g., anti-PD-1) to overcome EMT-induced resistance, ultimately bridging the gap from bench to bedside.

In conclusion, this study revealed that FKBP9 is a key EMT gene in a subpopulation of HCC cancer cells. FKBP9-mediated up-regulation and stabilization of SEPT6 activates the TGF-β pathway and drives HCC progression. Inhibition of FKBP9 by pinyonomycin effectively blocked the proliferation, migration, and EMT of HCC cells. These findings strongly support the exploration of FKBP9 as a promising therapeutic target for HCC.

## Supplementary Material

Supplementary materials and methods, figures and tables.

## Figures and Tables

**Figure 1:**
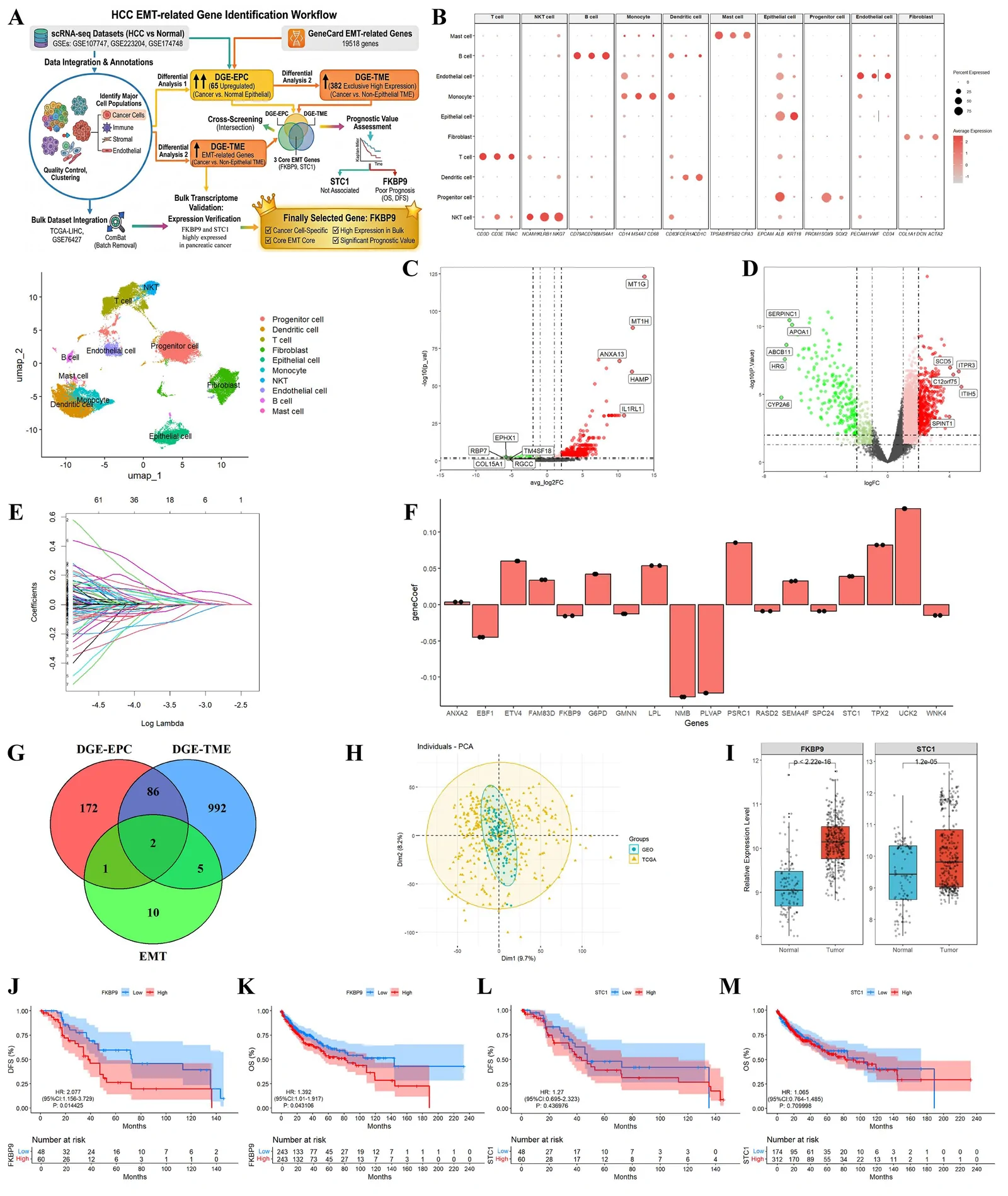
** Identification of FKBP9 as a cancer cell-specific EMT-related gene in HCC.** (A) Flowchart of the screening workflow for key genes associated with EMT in HCC; (B) UMAP plot showing cell clustering and annotation, as well as the expression profiles of canonical cell marker genes, based on the integrated scRNA-seq datasets; (C) Volcano plot of differentially expressed genes between HCC cancer cells and normal liver epithelial cells (DGE-EPC); (D) Volcano plot of differentially expressed genes between HCC cancer cells and non-epithelial cells in TME (DGE-TME); (E) Venn diagram showing the collection of EMT-related genes from GeneCards database; (F) LASSO-Cox regression screening for EMT-related prognostic genes, with 18 core genes included in the final model; (G) Venn diagram showing the intersection of DGE-EPC, DGE-TME and EMT-related genes, identifying FKBP9 and STC1 as core candidate genes; (H) Principal component analysis plot before and after batch effect correction of integrated TCGA-LIHC and GSE76427 bulk transcriptome datasets via ComBat method; (I) Box plot showing the expression of FKBP9 and STC1 in HCC tumor tissues and paired adjacent non-tumor tissues; (J-M) Kaplan-Meier survival curves for DFS and OS of HCC patients stratified by FKBP9 expression.

**Figure 2:**
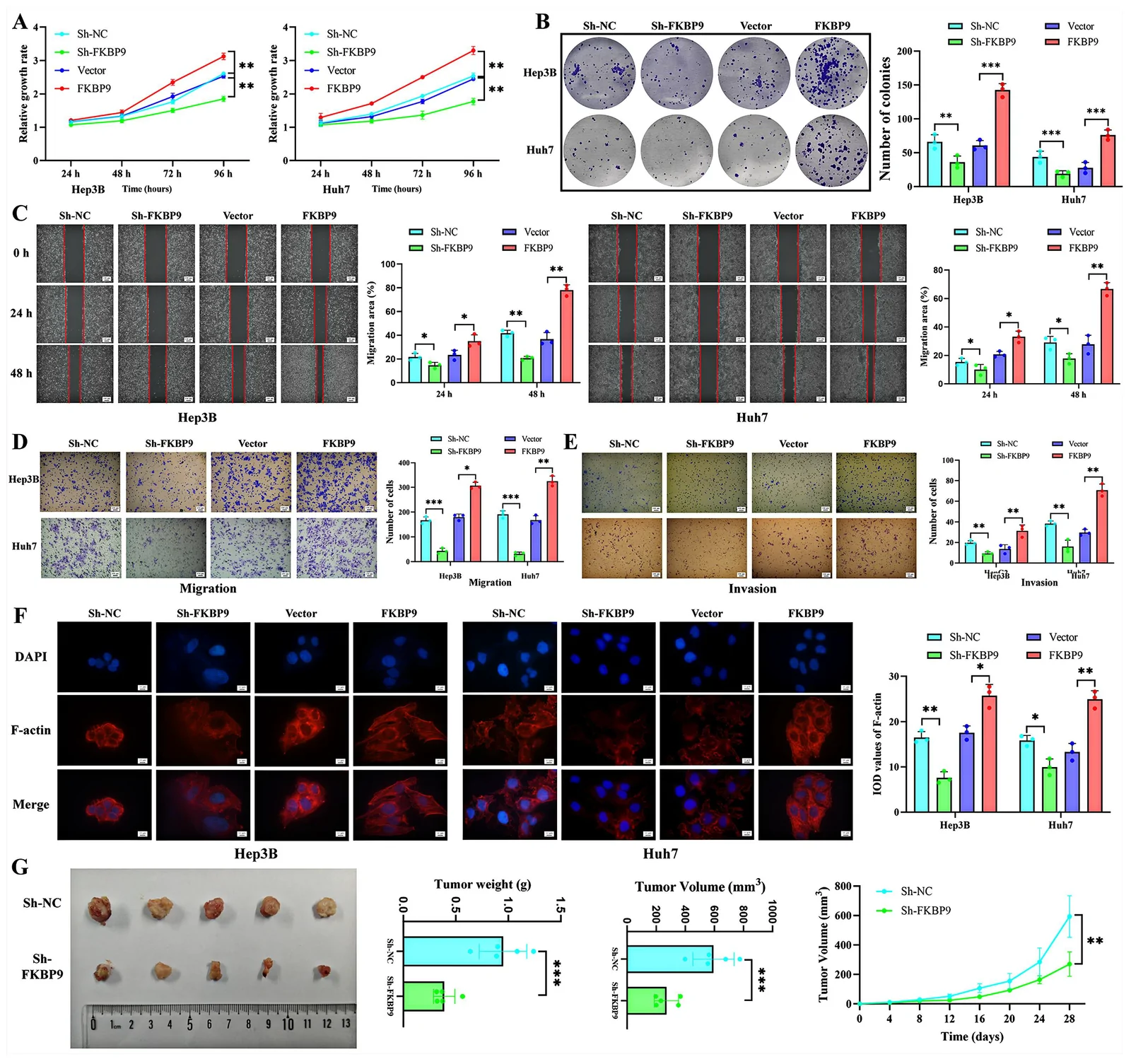
** FKBP9 promotes proliferation, migration, invasion and tumorigenesis of HCC cells.** (A-E) FKBP9 overexpression enhances the proliferation (A-B), migration (C-D), and invasion (E) HCC cells, and these promoting effects are reversed when FKBP9 is downregulated; (F) FKBP9 overexpression induced F-actin upregulation and a spindle-like morphology, suggesting that FKBP9 promotes the EMT process, and this effect is reversed upon FKBP9 downregulation; (G) Knockdown of FKBP9 inhibits the proliferation and tumorigenic capacity of subcutaneous xenografts in nude mice. *p <0.05.**p < 0.01. ***p < 0.001. NS, no significance.

**Figure 3:**
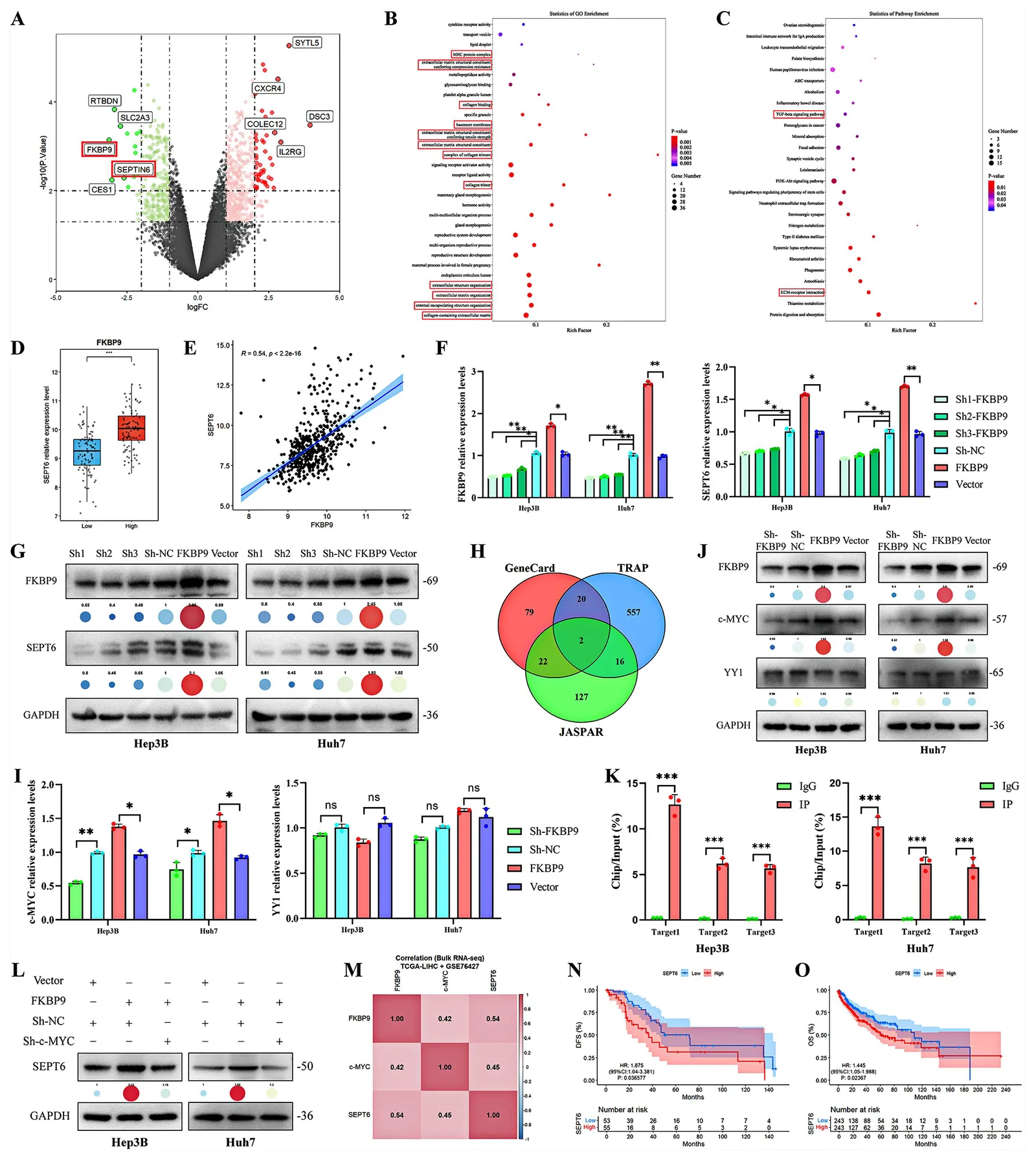
** FKBP9 promotes SEPT6 transcription via the transcription factor c-MYC.** (A) Volcano plot of differentially expressed genes (DEGs) from FKBP9 transcriptome sequencing; (B-C) GO and KEGG enrichment analyses of FKBP9-related DEGs, indicating the activation of the EMT process and TGF-β pathway; (D-E) SEPT6 and FKBP9 exhibit consistent expression patterns in HCC and show a significant positive correlation; (F-G) PCR and WB assays confirm that the expression level of SEPT6 in HCC cells is regulated by FKBP9; (H) Transcription factor prediction suggests that FKBP9 may promote SEPT6 expression through c-MYC and YY1; (I-J) PCR and WB analyses demonstrate that c-MYC expression increases with FKBP9 overexpression, whereas YY1 expression does not show such a change; (K) ChIP assay confirms the transcriptional regulation of SEPT6 by c-MYC; (L) Rescue experiments show that FKBP9 overexpression significantly upregulates SEPT6, while c-MYC knockdown reverses the elevated SEPT6 expression induced by FKBP9 overexpression; (M) Correlation analysis reveals a significant positive correlation among FKBP9, c-MYC and SEPT6 in HCC tissues; (N-O) SEPT6 is significantly associated with patient prognosis. *p <0.05.**p < 0.01. ***p < 0.001. NS, no significance.

**Figure 4:**
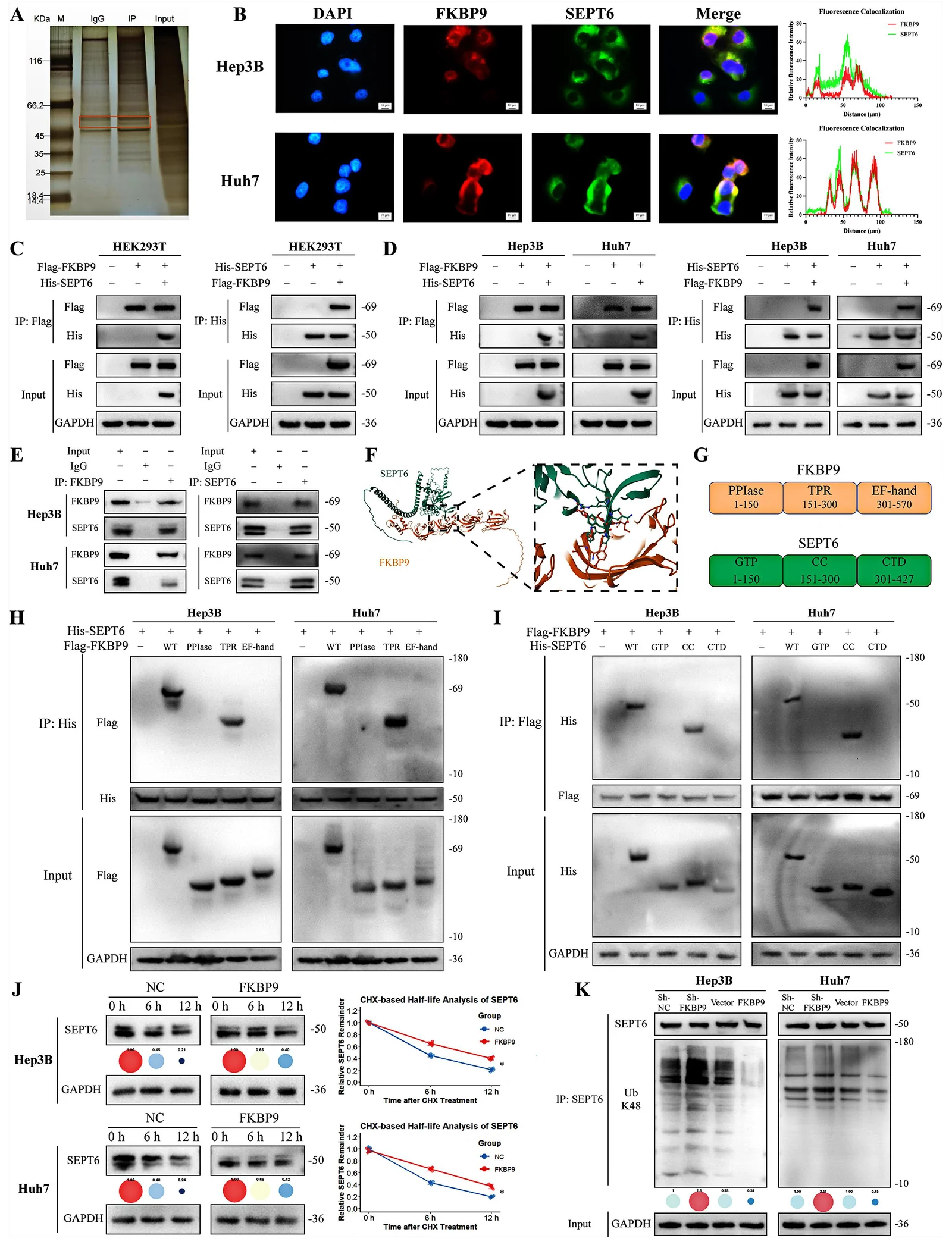
** FKBP9 directly binds to SEPT6 and enhances its protein stability.** (A) Silver staining of Flag-FKBP9 pull-down samples from mass spectrometry analysis shows a binding band at the ~50 kDa position (corresponding to SEPT6); (B) Fluorescence co-localization analysis indicates that SEPT6 and FKBP9 exhibit similar subcellular localizations in HCC cells; (C-D) Co-IP assays confirm that exogenous Flag-FKBP9 can directly bind to His-SEPT6 in HEK293T cells and HCC cells; (E) Co-IP assay verifies the direct binding between endogenous FKBP9 and SEPT6 in HCC cells; (F) The DMfold deep learning model predicts multiple binding sites between FKBP9 and SEPT6; (G-I) FKBP9 and SEPT6 were each divided into multiple truncation mutants based on their structural domains, where the TPR domain of FKBP9 and the CC domain of SEPT6 are the essential domains for their interaction; (J) CHX-based half-life analysis suggests that FKBP9 not only promotes SEPT6 expression but also enhances its protein stability; (K) FKBP9 knockdown significantly increased K48-linked polyubiquitination of SEPT6, while FKBP9 overexpression exerted the opposite effect. *p <0.05.

**Figure 5:**
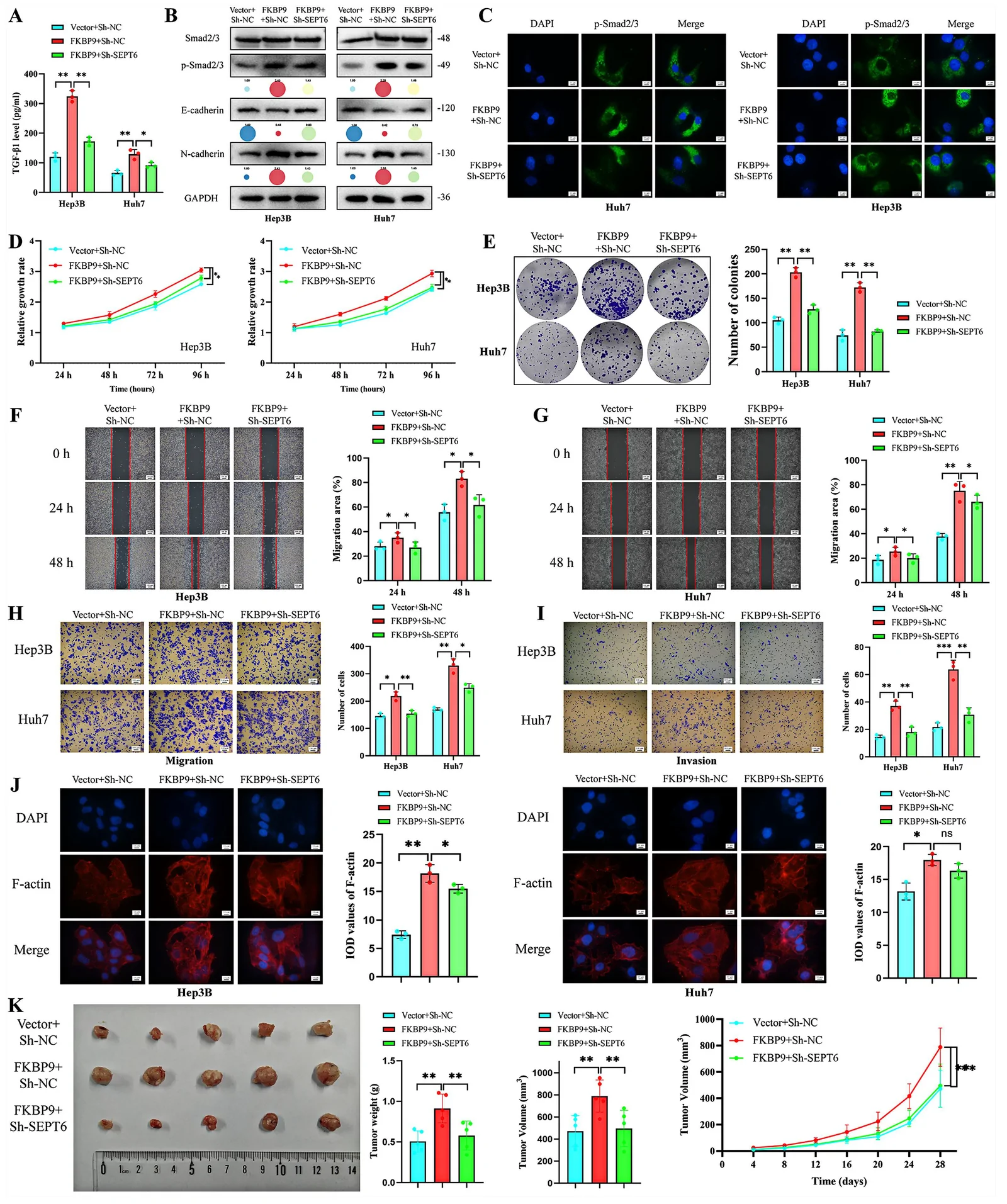
** FKBP9 activates the TGF-β pathway via SEPT6 to drive EMT and malignant progression of HCC.** (A) ELISA results showed that FKBP9 overexpression significantly increased the concentration of TGF-β1 in the supernatant of HCC cells, which was reversed by SEPT6 knockdown; (B-C) FKBP9 overexpression upregulated the level and nuclear translocation of p-Smad2/3, which was reversed by SEPT6 knockdown; (D-E) FKBP9 overexpression promoted the proliferation and clonogenic capacity of HCC cells, which was reversed by SEPT6 knockdown; (F-I) FKBP9 overexpression enhanced the migratory and invasive abilities of HCC cells, which was reversed by SEPT6 knockdown; (J) FKBP9 overexpression increased the expression of F-actin and induced a spindle-like mesenchymal morphological change in HCC cells, which was reversed by SEPT6 knockdown; (K) FKBP9 overexpression enhanced the tumorigenic capacity of HCC cells, which was reversed by SEPT6 knockdown. *p <0.05. **p < 0.01. ***p < 0.001. NS, no significance.

**Figure 6:**
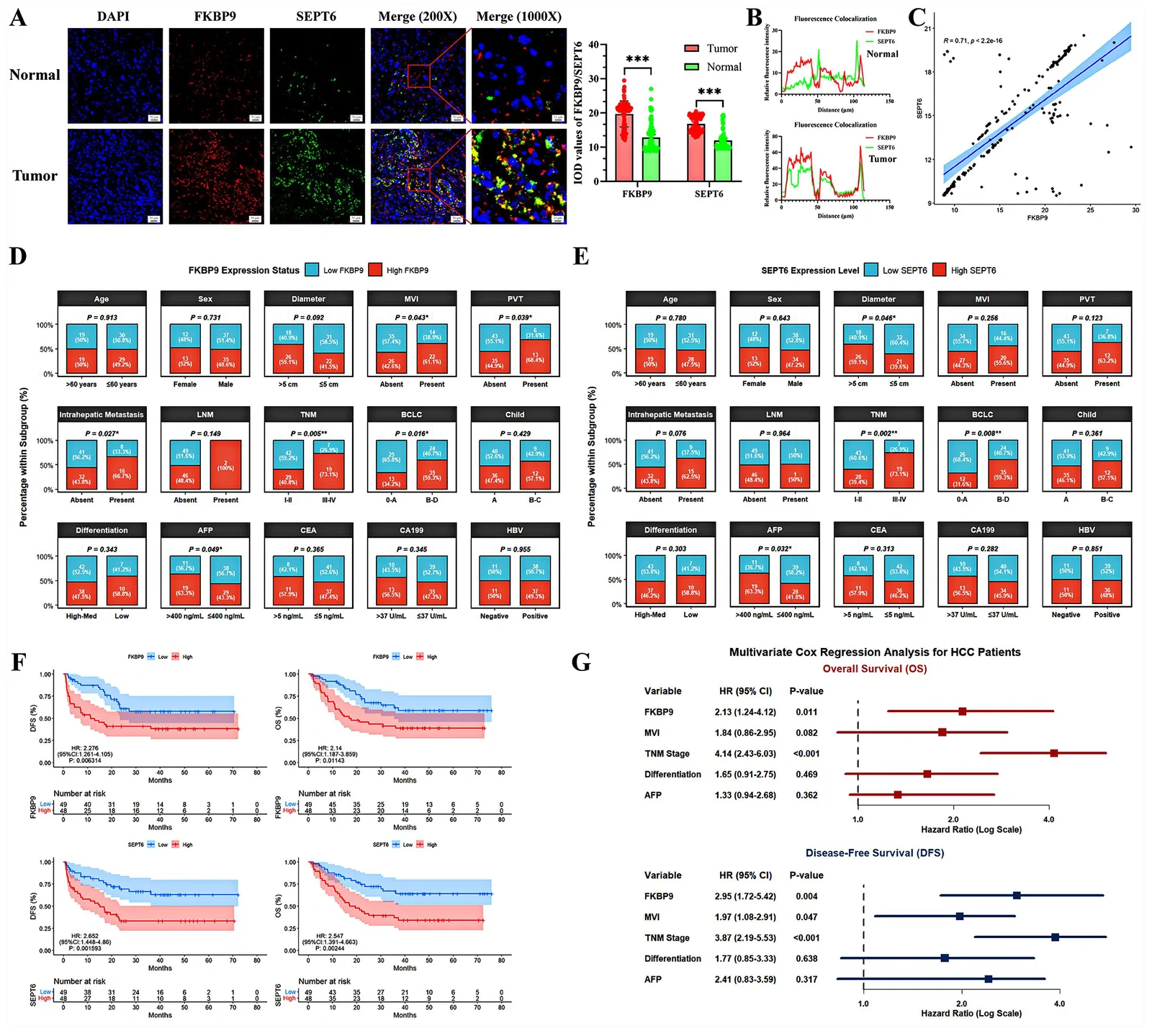
** Expression characteristics and prognostic value of FKBP9 and SEPT6 in clinical HCC tissues.** (A) Representative images of multiplex immunofluorescence staining of FKBP9 and SEPT6 in paired tumor tissues and adjacent non-tumor tissues from 97 HCC patients (left: 200×magnification; right: 1000×magnification). (B) Quantitative analysis of fluorescence co-localization of FKBP9 and SEPT6 in HCC tissues and adjacent non-tumor tissues. (C) Pearson correlation analysis of FKBP9 and SEPT6 protein expression levels in HCC tissues. (D-E) Comparison of distribution differences of key malignant clinicopathological features between patients with high and low expression of FKBP9 (D) and SEPT6 (E). (F) Kaplan-Meier survival curve for overall survival of HCC patients grouped by the median expression of FKBP9. (G) Forest plot of multivariate Cox regression analysis of indicators associated with overall survival of HCC patients. ***p < 0.001.

**Figure 7:**
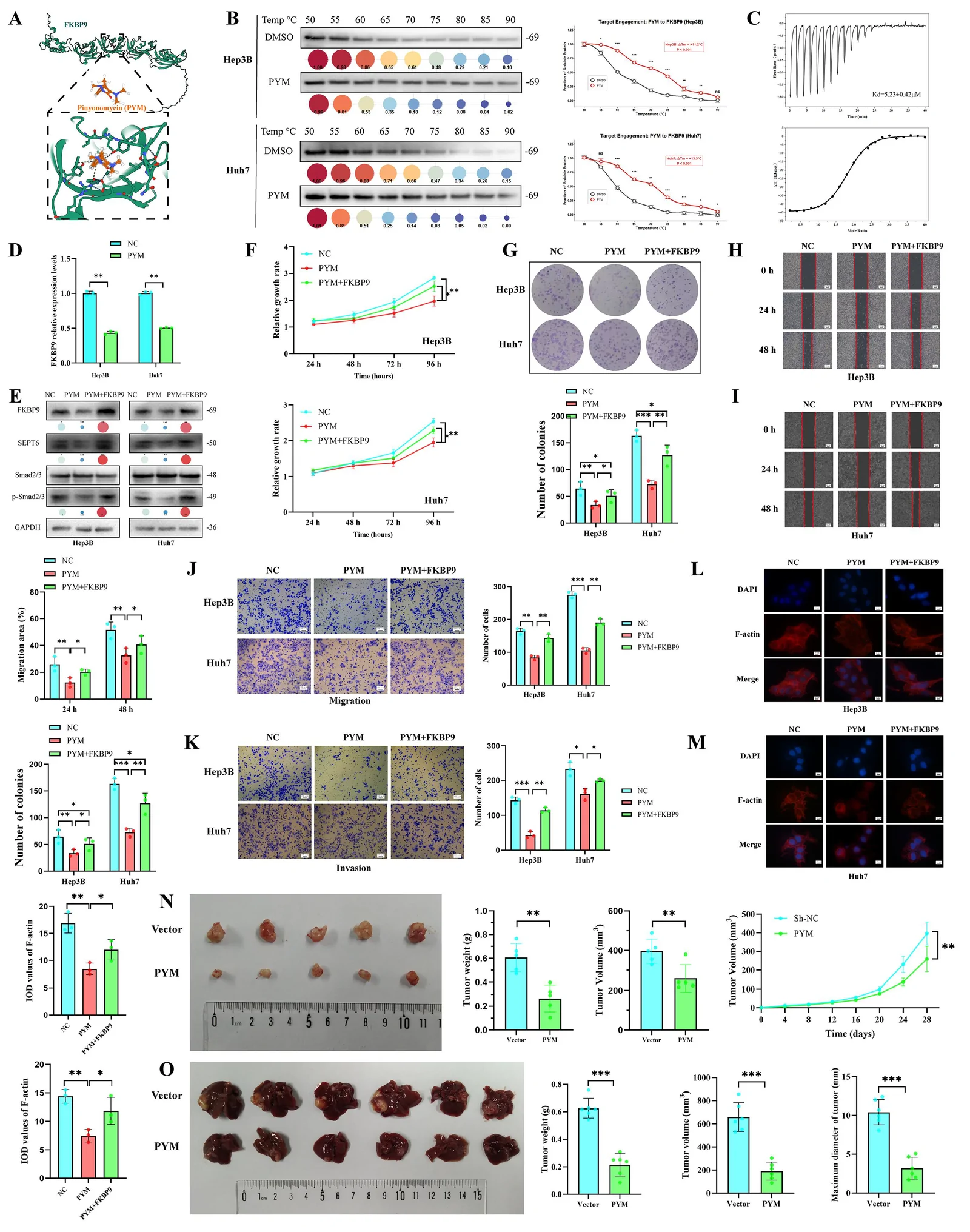
** FKBP9 inhibitor pinyonomycin inhibits hepatocellular carcinoma cell proliferation, invasion and EMT.** (A) The target drug pinyonomycin for FKBP9 was predicted by DSigDB, and the binding sites of pinyonomycin and FKBP9 were calculated by deep learning; (B) ITC assays revealed that pinyonomycin exhibited a distinct binding affinity to the recombinant FKBP9 protein; (C) CETSA assays demonstrated that pinyonomycin markedly enhanced the thermal stability of FKBP9 protein; (D-E) The addition of pinyonomycin inhibited the expression level of FKBP9/SEPT6 and TGF-β1 activation, while overexpression of FKBP9 effectively reversed this phenomenon; (F-M) The addition of pinyonomycin inhibited the proliferation (F-G), migration (H-J) and invasion (K) of HCC cells, while the expression of F-actin decreased and showed a rounded shape alteration (L-M), which was alleviated by overexpression of FKBP9. (N) HCC subcutaneous xenograft tumor model (n=5 per group): representative images of tumors, tumor weight statistics and tumor growth curves show that pinyonomycin treatment significantly inhibits subcutaneous tumor growth; (O) HCC orthotopic liver xenograft tumor model (n=6): representative images of liver tumors show that pinyonomycin treatment markedly suppresses the growth of orthotopic liver tumors. *p <0.05. **p < 0.01. ***p < 0.001. NS, no significance.

## Data Availability

Due to data privacy, the data in this study are available upon request to the corresponding author(s).
