# Supplementary material for: FKBP9 Drives Hepatocellular Carcinoma EMT by Regulating SEPT6 Transcription and Stability to Activate TGF-β Signaling
Source: Int J Biol Sci. 2026 Sep 11;22(15):8286–301. doi: 10.7150/ijbs.134156 (PMC13685609; doi:10.7150/ijbs.134156)
Supplement: Supplementary file 1 [file ijbsv22p8286s1.pdf]

Supplementary materials

Patients and cells

1. Public Dataset Collection

In this study, we integrated The Cancer Genome Atlas Liver Hepatocellular Carcinoma (TCGA-LIHC) dataset (transcriptome and clinical data from 371 HCC patients) and the GSE76427 dataset (transcriptome and clinical data from 167 HCC patients). After batch effect correction using the ComBat method, the merged dataset was applied for expression validation and prognostic correlation analysis of target genes. Meanwhile, single-cell RNA sequencing (scRNA-seq) datasets including GSE107747, GSE223204 and GSE174748 were integrated for cell subpopulation annotation and cell type-specific expression analysis of target genes.

2. Clinical Patient Cohort and Tissue Samples

This was a single-center retrospective cohort study, approved by the Ethics Committee of The Second Affiliated Hospital of Zhejiang University School of Medicine (Approval No. 2024-0397). Written informed consent was obtained from all enrolled patients, and the entire study was conducted in strict accordance with the Declaration of Helsinki.

Sample Collection and Processing: Within 10 minutes after surgical resection, tumor tissues and paired adjacent non-tumorous liver tissues were harvested separately. After rinsing with sterile normal saline to remove residual blood, the specimens were immediately fixed in 4% paraformaldehyde solution, followed by routine dehydration and paraffin embedding for subsequent mIF staining experiments. The pathological diagnosis of all specimens was confirmed via double-blind review by two senior pathologists.

Follow-up Protocol and Endpoint Definition: A standardized follow-up protocol combining outpatient and inpatient visits was implemented for all patients after surgery: follow-up was performed every 3 months for the first 2 postoperative years, every 6 months from the 3rd to the 5th postoperative year, and once annually thereafter. Follow-up assessments included serum tumor marker detection [alpha-fetoprotein (AFP), carcinoembryonic antigen (CEA), carbohydrate antigen 19-9 (CA19-9), etc.], abdominal imaging examinations (ultrasound, contrast-enhanced computed tomography/magnetic resonance imaging [CT/MRI]), and chest CT. Positron emission tomography-computed tomography (PET-CT) was performed when necessary to confirm tumor recurrence or

metastasis.

Definition of Follow-up Endpoints: Primary endpoint: Overall Survival (OS), defined as the interval from the date of radical hepatectomy to death from any cause. Secondary endpoint: Disease-Free Survival (DFS), defined as the interval from the date of radical hepatectomy to the first occurrence of tumor recurrence, distant metastasis, or death from any cause.

Inclusion criteria: Pathologically confirmed primary hepatocellular carcinoma after surgical resection; Received curative hepatectomy, with no preoperative neoadjuvant therapy (including radiotherapy, chemotherapy, targeted therapy, immunotherapy, or interventional therapy); Complete clinicopathological data and postoperative follow-up information available; Paired tumor tissue and adjacent non-tumor liver tissue available with qualified sample quality for multiplex immunofluorescence staining.

Exclusion criteria: Received any preoperative neoadjuvant therapy, transarterial chemoembolization, or systemic anti-tumor therapy; Concurrent with other primary malignant tumors; Severe dysfunction of vital organs (heart, lung, liver, kidney), autoimmune diseases, or hematological diseases; Incomplete clinicopathological or follow-up data; Death within 30 days after surgery.

3. Cell Lines

Human HCC cell lines Hep3B, Huh7, and human embryonic kidney 293T (HEK293T) cells were purchased from the American Type Culture Collection (ATCC, Manassas, VA, USA). Hep3B and Huh7 cells were routinely cultured in Dulbecco's Modified Eagle Medium (DMEM) supplemented with 10% fetal bovine serum (FBS) and 1% penicillin-streptomycin, in a humidified incubator at 37°C with 5% CO_2. Cells were passaged every 2–3 days, and all experiments were performed using cells in the logarithmic growth phase. All cell lines were authenticated via short tandem repeat (STR) profiling and confirmed to be free of mycoplasma contamination.

Cell transfection

Cell Transfection & Establishment of Stable Cell Lines

Stable knockdown or overexpression of target genes was achieved by lentiviral delivery system. The shRNA target sequences for human FKBP9, MYC, SEPT6, GAPDH (positive control) and scramble negative control (sh-NC) were designed and chemically synthesized, with full sequences listed in Table S1. The shRNA fragments were cloned into the lentiviral pLKO.1-TRC vector (ampicillin resistance, puromycin screening marker) for stable gene knockdown. All recombinant plasmids were

verified by Sanger sequencing to ensure 100% sequence accuracy.

Lentivirus Packaging and Titer Determination

Lentivirus particles were packaged in HEK293T cells using the calcium phosphate transfection method. Briefly, HEK293T cells were seeded into 6-well plates at a density of 1×10^6 cells/well 24 h before transfection, and transfection was performed when cell confluence reached 70%–80%. For each well, the transfection complex was prepared with a 2nd generation lentiviral packaging system: 2.5 μg of recombinant pLKO.1 plasmid, 1.875 μg of psPAX2 packaging plasmid, 0.625 μg of pMD2.G envelope plasmid, and calcium phosphate transfection kit (CAPHOS-1KT, Sigma-Aldrich, USA) following the manufacturer's instructions. The transfection complex was incubated at room temperature for 20 min and then added dropwise to the cell culture medium.

Cell supernatants were harvested at 48 h and 72 h post-transfection, respectively, and filtered through a 0.45 μm sterile PVDF filter to remove cell debris. The lentivirus titer was determined by the limiting dilution method combined with RT-qPCR, and only virus preparations with a titer $\geq 1\times10^8$ TU/mL were used for subsequent cell infection experiments.

Lentiviral Infection and Antibiotic Screening

Hep3B and Huh7 cells were seeded into 6-well plates at a density of 2×10^5 cells/well 24 h before infection, and infection was performed when cell confluence reached 50%–60%. The original culture medium was discarded, and fresh complete medium containing the corresponding lentivirus and 8 μg/mL Polybrene (TR-1003, Sigma-Aldrich, USA) was added. The optimal multiplicity of infection (MOI) was determined by pre-experiments: MOI=10 for Hep3B cells, MOI=15 for Huh7 cells. After incubation at 37℃ in a 5% CO_2 humidified incubator for 12 h, the virus-containing medium was replaced with fresh complete medium without Polybrene for further culture.

The working concentrations of screening antibiotics were determined by lethal concentration pre-experiments: puromycin (2 μg/mL for Hep3B cells, 2.5 μg/mL for Huh7 cells) for pLKO.1 vector-transduced cells. At 48 h post-infection, the medium was replaced with fresh complete medium containing the corresponding screening antibiotic, and the medium was refreshed every 2 days. The screening was continued for 7 days until all cells in the blank control group (uninfected cells treated with the same concentration of antibiotics) were completely dead. After screening, cells were maintained in complete medium with 1/2 of the screening concentration of antibiotics for subsequent experiments.

Monoclonal Cell Line Construction and Validation

Monoclonal stable cell lines were established by limiting dilution method. Briefly, the polyclonal cell pool after antibiotic screening was digested with trypsin and resuspended to a single-cell suspension with a density of 5 cells/mL. The cell suspension was seeded into 96-well plates at 100 μL/well to ensure that the number of cells per well was ≤1. The plates were incubated in a 37℃, 5% CO_2 humidified incubator for 14 days, and wells containing only a single cell clone were marked daily under a microscope. When the monoclonal cells grew to 80% confluence, they were digested with trypsin and subcultured for expansion.

The knockdown/overexpression efficiency of target genes in monoclonal cell lines was verified by both RT-qPCR and Western blot. Monoclonal cell lines with target gene knockdown efficiency >80% or overexpression efficiency >5-fold were selected for subsequent in vitro and in vivo functional experiments.

PCR and western blot

Total RNA was isolated using TRIzol reagent following the manufacturer's protocol. Reverse transcription was conducted with PrimeScript RT Enzyme (Takara Biotechnology). Real-time PCR amplification was carried out on a ABI7900HT Real-TimePCR system (AppliedBiosystemsLifeTechnologies, FosterCity, CA, USA) using SYBR® Premix Ex Taq™ II. Cycling conditions were as follows: 95°C for 10 seconds, followed by 40 cycles of 95°C for 5 seconds and 60°C for 31 seconds. Relative gene expression was calculated using the 2-ΔΔCT method, with β-Actin serving as the internal control. Primer sequences are listed in Table S2.

Homogenization was performed with cell lysis buffer (P0013B, Beyotime, China). The supernatant was collected as protein samples. Protein content was determined using a BCA protein assay kit (P0009, Beyotime, China). Aliquots of protein samples were separated by denaturing 10% SDS-PAGE and transferred to polyvinylidene difluoride (PVDF) membranes. The membrane was then incubated with a specific primary antibody at 4°C overnight with stirring. The membrane was then incubated with horseradish peroxidase (HRP)-conjugated secondary antibody (1:1000) for 60 min at RT. Finally, protein bands were detected using an enhanced chemiluminescence (ECL) protein blotting detection system (GE healthcare, Amersham, UK).

Antibody information:FKBP9 (18073-1-AP, Proteintech, China, 1:1000), FLAG (66008-4-Ig, Proteintech, China, 1:20000), SEPT6 (12805-1-AP, Proteintech, China, 1:1000), GAPDH (60004-1-Ig, Proteintech, China, 1:50,000), c-MYC (10828-1-AP, Proteintech, China, 1:5000), YY1 (82712-3-RR,

Proteintech, China, 1:5000), SMAD2/3 (YT4332, Immunoway, China, 1:2000), p-SMAD2/3 (YP0362, Immunoway, China, 1:1000) E-Cadherin (20874-1-AP, Proteintech, China, 1:50,000), and N Cadherin (22018-1-AP, Proteintech, China, 1:10000).

We performed systematic optical density quantification using ImageJ software on all biological replicates of the Western blot experiments in this study. All quantifications used GAPDH as the internal reference protein to calculate the relative gray values of the target protein in different treatment groups (for quantification of phosphorylation levels, total protein expression was used as the baseline/internal reference). Quantification results from no fewer than three independent biological replicates were included in each experiment to ensure data reproducibility and statistical rigor.

Cell counting kit-8 assay

HCC cells of each designated nomenclature group were transferred into 96-well plates (1×10^3 cells/well), and cell counting kit-8 (CCK-8) solution (beyotime, CO048M, China) was added to each well at 24, 48, 72 and 96 h, respectively, followed by 3 h of incubation. The absorbance of each well was recorded at 450 nm using a microplate reader (BioTek, Winooski, VT, USA).

Cell colony formation assay

In the cell colony formation assay, Hep3B and Huh7 cells were cultured in 6-well plates at a concentration of 800 cells/well. Then the medium was changed every two days and the cells were allowed to grow for a fortnight. The cells were washed with PBS and fixed, and stained with 1% crystal violet solution for 15 min. The excess stain was rinsed with PBS, and then the images were captured with a microscope.

Scratch experiment

Trypsin digests the cells and prepares cell suspension. Cell counting and plate spreading (six-well plate), to ensure that the density of each group of cell spreading is consistent, generally inoculated in the wells of about $5\text{-}10*10^5$ cells. (Need to set up a control group). In the six-well plate lateral marking 5 horizontal marking line, to be full of cells, with a ruler then the use of 20 ul gun tip perpendicular to the plate and line to create three cell scratches, so that the scratches intersect with the marking line. After the scratches are completed, take a picture with a microscope as a 0 h control. The old medium was aspirated, and then the cells were washed three times with sterile PBS (added slowly against the

wall) to wash away the scratched cells so that the gaps left behind were visible to the naked eye. Then add fresh serum-free or low serum (<2%) medium. Put the cells into a 37°C, 5% CO2 incubator for culture. The width of the scratch was then observed and photographed at appropriate time points.

Transwell

Digest the cells, resuspend the cells with serum-free medium, count the cells (blow and mix before each counting, and take the liquid immediately for counting), adjust the cell density to 1-10×10^5/mL, and inoculate 100-300ul of cell suspension into the cell chamber above the transwell (generally 2-5×10^4 cells can be added, and the full-grown flasks are about 5×106, so one-tenth of them will be closer). (It will be close to the original culture medium), add 600ul of complete culture medium with 10% concentration to the lower well plate, and incubate in the incubator for 24 hours. Discard the original culture medium, wash with PBS once, add 4% paraformaldehyde solution to each well (similar to the culture medium, no more than the inside and outside of the chamber), and fix it for 20 minutes at room temperature. Discard the fixation solution, wash with tap water once (dry before staining), and add 0.1% (1 mg) of the total cell suspension to the upper well of the transwell. Add 0.1% (1 mg/mL) crystal violet solution to each well, recover the crystal violet after 15 minutes of staining, wash once with tap water (PBS), and wipe away the non-migrated cells inside the chamber with a cotton swab. Remove the membrane at the bottom of the chamber with tweezers, with the bottom side facing upwards, transfer it to a slide and seal the slides with a neutral resin adhesive. The transwell without matrix gel is for the migration experiment. Transwells containing matrix gel are invasion experiments.

Cytoskeleton staining

(1) Wash the cells 3 times with PBS. (2) Cells were fixed with PBS solution containing 4% formaldehyde (without methanol) for 20 min at room temperature. (3) Cells were washed 3 times with PBS. (4) The cells were permeabilized with PBS solution containing 0.4% TritonX-100 for 10 min at room temperature. (5) The cells were washed 3 times with PBS. (6) Dilute 1-5 μL of fluorescently labeled Ghost Pen Cyclic Peptide Reservoir Solution with 200 μLPBS, add to one coverslip or well, incubate for 20 min at room temperature, and stain. Note: The chromosome product can be adjusted according to the sample. To avoid volatilization of the staining solution during incubation, the coverslip can be placed in a sealed container. Wash the cells twice with PBS. Restain the nuclei using 200 μLDAPI solution (concentration: 100 nM) for about 30 s. Observe by fluorescence microscopy. The labeled ghost

pen cyclic peptide is very light and stable and samples can be imaged in PBS, but for best results, they can also be viewed with an anti-fluorescence quencher.

Co-immunoprecipitation (COIP) assay

Whole cells were lysed into NP-40 lysis buffer (20 mMTris-HClpH8.0, 137 mMNaCl, 10% glycerol) containing 1% NP-40 and protease inhibitor cocktail (P1005, Beyotime, China) in an ice bath for 10 min. The mixture was sonicated at 30% intensity (10 times for 2 s on ice). The supernatant was collected and incubated with the applicable beads for 2-3 h at 4°C, then rinsed three times with ice-cold PBS. Finally, samples were boiled in an SDS loading buffer and analyzed by Western blotting.

Chromatin immunoprecipitation (ChIP) assay

The ChIP was performed according to the ChIP kit (Bes5001, bersinbio, China): Hep3B and Huh7 cells were collected and sonicated to generate DNA fragments of 200 to 500 bp. Lysates were then immunoprecipitated with anti-c-MYC, YY1, or IgG antibodies overnight. The immunoprecipitated DNA was extracted and analyzed by qPCR.

c-MYC binding site prediction and validation: The canonical c-MYC binding motifs in the SEPT6 promoter region (2000 bp upstream to 500 bp downstream of the TSS of SEPT6 MANE transcript ENST00000394610.7, hg38) were predicted using the JASPAR 2024 database with the c-MYC position weight matrices (MA0147.3 and MA0147.4). A total of 5 high-confidence binding sites (relative score > 0.85) were identified, among which 3 representative sites in the distal, middle and proximal promoter regions were selected for ChIP-qPCR validation. The detailed information of the 3 target sites is shown in Table S3.

Dual-Luciferase Reporter Assay

The wild-type (WT) SEPT6 promoter fragment covering the region from 2000 bp upstream to 500 bp downstream of the transcription start site (TSS) was cloned into the pGL3-Basic luciferase reporter vector (Promega, Madison, WI, USA). Site-directed mutagenesis was performed to mutate the canonical c-MYC binding motifs in the SEPT6 promoter to generate mutant-type (MUT) reporter constructs. The pRL-TK vector (Promega) encoding Renilla luciferase was co-transfected as an internal reference for normalization of transfection efficiency. All plasmids were verified by Sanger sequencing and extracted using an endo-free plasmid maxiprep kit.

Human HCC Hep3B and Huh7 cell lines were obtained from the American Type Culture Collection (ATCC, Manassas, VA, USA) and cultured in Dulbecco's Modified Eagle Medium (DMEM) supplemented with 10% fetal bovine serum (FBS) and 1% penicillin-streptomycin. Cells were seeded into 96-well white opaque plates at a density of 2×10^4 cells per well 18–24 h before transfection. Transfection was performed using Lipofectamine 3000 reagent (Invitrogen, Carlsbad, CA, USA) following the manufacturer's protocol, with a fixed 10:1 mass ratio of firefly luciferase reporter plasmid to pRL-TK Renilla reference plasmid. The total amount of plasmid per well was kept consistent across all groups by supplementing with the corresponding empty vector.

At 24–48 h post-transfection, firefly and Renilla luciferase activities were detected sequentially using the Dual-Luciferase Reporter Assay System (Promega) with a multifunctional microplate reader (BioTek, Winooski, VT, USA). The relative luciferase activity was calculated as the ratio of firefly luciferase luminescence value to Renilla luciferase luminescence value for each well. All experiments were performed with at least three independent biological replicates, and each treatment was set up in triplicate technical replicates. Statistical analysis was performed using Student's t-test for two-group comparisons and one-way ANOVA followed by Tukey's post-hoc test for multi-group comparisons at a single time point, consistent with the global statistical analysis protocol described in the Methods section. $P < 0.05$ was considered statistically significant..

Dosing experiment

Half-life analysis: CHX (66-81-9, GLPBIO, China) at a concentration of 100 μg/mL was added to the cell culture medium, and cellular proteins were collected at 0 h, 6 h, and 12 h after drug addition for quantitative analysis. Pinyonomycin: Cell experiments were performed by adding pinyonomycin (55658-47-4, GLPBIO, China) at a concentration of 1 μg/mL to the cell culture medium for 24 h.

For in vitro experiments, pinyonomycin (CAS: 55658-47-4, GLPBIO, China) was used at a final working concentration of 1 μg/mL. This concentration was established based on the 72 h half maximal inhibitory concentration (IC50) for cell proliferation in Hep3B and Huh7 cells, which was experimentally determined in the present study. After 24 h of drug treatment, cells were harvested for subsequent functional assays and molecular biological analyses.

For in vivo animal experiments, pinyonomycin was administered via intraperitoneal (i.p.) injection at a dose of 10 mg/kg every other day. This dosage was determined primarily based on the in vitro IC50 measured in our study, as well as the safe and effective dose range of this compound for intraperitoneal

injection in mice reported in previously published authoritative peer-reviewed literature.

Tumor xenograft experiments

1. Basic Information of Experimental Animals

1.1 Strain and Qualification

Specific pathogen-free (SPF)-grade BALB/c nude mice were purchased from Beijing Vital River Laboratory Animal Technology Co., Ltd.. All animals had clear genetic backgrounds, no specific pathogen infection, and were provided with complete animal quality certification.

1.2 Inclusion Criteria

Male nude mice were selected to eliminate the interference of sex hormone levels on the tumorigenicity of hepatocellular carcinoma (HCC). Mice were included in the formal experiment if they met the following criteria: aged 4–5 weeks at enrollment with an age difference ≤ 3 days within the same batch; body weight ranging from 18–22 g with a body weight difference ≤ 2 g within the same batch; no abnormal signs and stable body weight gain during the acclimatization period.

1.3 Housing Environment and Management

Facility Qualification: All animals were housed in the SPF-grade laboratory animal barrier system of Taizhou Hospital of Zhejiang University School of Medicine throughout the experiment.

Environmental Parameters: The housing environment was maintained at a constant temperature of 22 ± 2 ℃, constant humidity of 50 ± 10%, air exchange rate ≥ 15 times/h, 12 h/12 h light-dark cycle (light on at 8:00 and off at 20:00), and environmental noise ≤ 60 dB.

Feeding Management: Animals were housed in individually ventilated cages (IVC) with no more than 5 mice per cage. Autoclaved corn cob bedding was used and changed twice a week. Animals had ad libitum access to ^{60}Co-irradiated sterilized SPF-grade maintenance diet for nude mice and autoclaved ultrapure water. Cages and water bottles were autoclaved before use and changed once a week.

Acclimatization: After arrival, animals were acclimatized in the barrier environment for 7 days. Body weight, mental state, food and water intake were monitored daily, and animals with no abnormalities were included in the experiment.

1.4 Personnel Qualification

All operators involved in the animal experiment received professional training for laboratory animal practitioners and obtained official qualification certificates. All operators completed standardized operation procedure (SOP) training before the experiment to ensure operation consistency

and minimize manual bias.

2. Detailed Methods for Tumor Model Establishment

2.1 Cell Suspension Preparation

The human HCC Hep3B cell line used in the experiment was confirmed to be authenticated via short tandem repeat (STR) profiling and negative for mycoplasma contamination. According to the experimental purposes, Hep3B cell lines stably transfected with sh-NC (short hairpin RNA negative control), sh-FKBP9, empty vector, FKBP9 overexpression plasmid, and FKBP9 overexpression plasmid + sh-SEPT6 were constructed respectively.

Target cells in the logarithmic growth phase were collected. The culture medium was discarded, and cells were washed twice with sterile phosphate-buffered saline (PBS). Cells were digested with 0.25% trypsin-EDTA until they rounded up and detached, and the digestion was terminated by complete Dulbecco's Modified Eagle Medium (DMEM) supplemented with 10% fetal bovine serum (FBS).

The cell suspension was centrifuged at 1000 rpm for 5 min at 4 °C. The supernatant was discarded, and the cell pellet was resuspended and washed twice with sterile PBS. The cell concentration was adjusted to the required inoculation density, and the suspension was kept on ice. Inoculation was completed within 30 min after preparation.

2.2 Establishment of Subcutaneous Xenograft Tumor Model

Anesthesia

Isoflurane inhalation anesthesia was performed with an induction concentration of 3% and a maintenance concentration of 1.5%. Complete loss of corneal reflex and righting reflex was confirmed in the animals before the operation.

Inoculation

The skin of the right back of nude mice was disinfected with 75% medical alcohol. A 1 mL sterile syringe with a 29G needle was used to aspirate the cell suspension, which was slowly injected subcutaneously into the right back at an inoculation dose of 1×10^6 cells / 100 μL PBS per mouse. The needle was slowly withdrawn after injection, and the injection site was pressed with a sterile cotton swab for 5 s to confirm no leakage or bleeding. The animals were then returned to the cage and continued to be housed after full recovery from anesthesia.

Tumor Formation Monitoring

The general condition of the animals was observed daily after inoculation. Palpation of subcutaneous nodules was initiated on day 7 post-inoculation, and subsequent experimental procedures

were performed after tumor formation was confirmed.

2.3 Establishment of Orthotopic Liver Xenograft Tumor Model

Preoperative Preparation

Animals were fasted for 12 h before surgery with ad libitum access to water. All surgical instruments were autoclaved, and aseptic techniques were strictly implemented throughout the procedure.

Anesthesia and Laparotomy

Isoflurane inhalation anesthesia was administered (induction concentration of 3%, maintenance concentration of 1.5%). Animals were fixed in the supine position on a 37 ℃ constant-temperature operating table. After abdominal hair removal, the surgical area was disinfected alternately with 75% alcohol and iodophor for 3 times. A ~1 cm midline abdominal incision was made, and the skin, abdominal wall muscles and peritoneum were incised layer by layer to expose the liver. The left lobe of the liver was gently exposed with sterile saline-moistened gauze.

Inoculation

A 33G microsyringe was used to aspirate the cell suspension, which was slowly injected into the subcapsular space of the left liver lobe at an inoculation dose of 5×10^5 cells / 50 μL PBS per mouse, with an injection rate of 10 μL/min. The needle was retained for 10 s after injection, then slowly withdrawn. The injection site was pressed with sterile gelatin sponge for 30 s to confirm no bleeding or leakage, and the liver was gently returned to the abdominal cavity.

Postoperative Care

The abdominal wall muscles and skin were sutured layer by layer with 4-0 absorbable sutures, and the incision was disinfected with iodophor. After surgery, animals were placed on a 37 ℃ constant-temperature heating pad until full recovery from anesthesia. The incision healing, mental state and food intake were monitored daily for 3 days after surgery, and intramuscular injection of penicillin (200,000 U per mouse per day) was administered to prevent infection.

3. Experimental Grouping and Treatment Protocols

Three independent in vivo experiments were performed in this study, with the grouping and treatment protocols as follows:

3.1 Experiment 1: Effect of FKBP9 on the In Vivo Tumorigenic Capacity of HCC

Grouping: sh-NC negative control group, sh-FKBP9 knockdown group, with n=5 mice per group.

Treatment: Mice were inoculated with corresponding stably transfected Hep3B cells to establish

the subcutaneous xenograft tumor model. The experimental cycle was 28 days. Animals were euthanized at the experimental endpoint, and tumor tissues were harvested.

3.2 Experiment 2: Mediating Role of SEPT6 in FKBP9-Driven HCC Progression (In Vivo Rescue Experiment)

Grouping: Vector + sh-NC negative control group, FKBP9 + sh-NC overexpression group, FKBP9 + sh-SEPT6 rescue group, with n=5 mice per group.

Treatment: Mice were inoculated with corresponding stably transfected Hep3B cells to establish the subcutaneous xenograft tumor model. The experimental cycle was 28 days. Animals were euthanized at the experimental endpoint, and tumor tissues were harvested.

3.3 Experiment 3: In Vivo Anti-Tumor Efficacy Validation of Pinyonomycin Against HCC

Subcutaneous model grouping: Vehicle control group (normal saline + 1% DMSO), Pinyonomycin treatment group, with n=5 mice per group.

Treatment: The subcutaneous xenograft model was established by inoculation of Hep3B cells. Administration was initiated when the tumor volume reached approximately 100 mm^3. Pinyonomycin was administered via intraperitoneal injection at a dose of 10 mg/kg every other day. The control group was given an equal volume of vehicle with the same administration route and frequency.

Orthotopic model grouping: Vehicle control group, Pinyonomycin treatment group, with n=6 mice per group.

Treatment: The orthotopic liver xenograft tumor model was established. Administration was initiated on day 7 after surgery, with the same dose, route and frequency as the subcutaneous model, for 14 consecutive days. Animals were euthanized at the experimental endpoint, the liver was completely dissected, and tissue samples were harvested.

4. Basis for Sample Size Determination

Sample size was determined by power analysis. Two-sided Student's t-test (for two-group comparison) or one-way analysis of variance (ANOVA, for multi-group comparison) was adopted, with a statistical power (1-β) of 0.8 and a significance level α of 0.05. Based on the effect size of intergroup differences from previous similar in vivo HCC experiments, the calculation results showed that a minimum of 5 mice per group for the subcutaneous model and 6 mice per group for the orthotopic model were required to meet the requirements of intergroup statistical difference test.

The final sample size was set at n=5 per group for the subcutaneous model, which fully met the statistical power requirements; n=6 per group for the orthotopic model, which fully met the statistical

power requirements. Meanwhile, the 3R principles (Reduction, Replacement, Refinement) of laboratory animal welfare were strictly followed, and the number of experimental animals was minimized on the premise of ensuring statistical power.

Animal Exclusion Criteria: Animals that died due to non-experimental factors, failed to form tumors, or developed severe infection during the experiment were excluded and not included in the final statistical analysis.

5. Tumor Monitoring and Index Measurement

Measurement Specification: During the experiment, animal body weight was measured using an electronic balance every 3 days, and the tumor long diameter (L) and short diameter (W) were measured using a vernier caliper with an accuracy of 0.02 mm. All measurements were performed by the same experimenter to reduce measurement bias.

Tumor Volume Calculation Formula: Tumor volume V (mm^3) = (long diameter L × short diameter W^2) / 2, which is the internationally accepted standard formula for calculating the volume of in vivo xenograft tumors.

Sample Collection: After the animals were euthanized at the experimental endpoint, the tumor tissues were completely dissected, and the wet weight of the tumors was weighed with an electronic balance. The tissues were snap-frozen in liquid nitrogen and stored in a -80 ℃ ultra-low temperature freezer for subsequent molecular biological assays.

6. Humane Endpoint and Euthanasia Operation Specification

6.1 Clear Definition of Humane Endpoint

The general condition of the animals was observed twice daily (8:00 and 20:00) during the experiment. Any of the following conditions was defined as reaching the humane endpoint, and the experiment for the corresponding animal was terminated immediately with euthanasia performed to avoid unnecessary stress and pain in the animals throughout the process:

Tumor volume exceeds 2000 mm^3, or the long diameter of the tumor exceeds 15 mm (complying with the ethical requirements for the maximum volume limit of subcutaneous xenograft tumors);

The tumor is ulcerated, bleeding, infected or necrotic, or the growth location affects the animal’s basic physiological activities such as feeding, drinking, locomotion and defecation;

Animal body weight decreases by more than 20% compared with the initial body weight of the experiment, or body weight continues to decrease for 3 consecutive days without a rebound trend;

The animal presents severe painful manifestations such as severe lethargy, hunched posture,

dyspnea, dyskinesia, and limb paralysis;

Severe infection, dehiscence of the surgical incision, or prolapse of abdominal organs, or other serious complications evaluated by a licensed veterinarian that require termination of the experiment.

6.2 Euthanasia Operation Specification

Euthanasia Method: Gradual CO_2 inhalation method, which complies with the 2020 Edition of the American Veterinary Medical Association (AVMA) Guidelines for the Euthanasia of Animals.

Operation Procedure: Animals were placed in a dedicated euthanasia chamber, and CO_2 was introduced at a flow rate of 20% of the chamber volume per minute. After the animal's breathing and heartbeat completely ceased, CO_2 infusion was continued for an additional 10 min. Animal death was confirmed by the absence of heartbeat on palpation, pupil dilation, and loss of corneal reflex before sample collection was performed.

7. Ethical Compliance Statement

All animal experimental protocols in this study were reviewed and approved by the Animal Research Ethics Committee of Taizhou Hospital of Zhejiang University School of Medicine (Ethics Approval No. T8Y-2025069). The entire experimental procedure was strictly conducted in accordance with the Animal Research: Reporting of In Vivo Experiments (ARRIVE) 2.0 Guidelines, the National Institutes of Health (NIH) Guide for the Care and Use of Laboratory Animals, and the Guidelines for Welfare and Ethical Review of Laboratory Animals of the People's Republic of China. The welfare of experimental animals was fully guaranteed throughout the study.

Translation Compliance Notes for SCI Submission

Term Standardization: All terms adopt the internationally accepted standard expressions in the field of oncology and laboratory animal science, such as humane endpoint (not humanitarian endpoint), subcutaneous/orthotopic xenograft tumor, ad libitum access, snap-frozen, etc., which fully comply with the norms of top journals in hepatology and oncology.

Abbreviation Specification: All professional abbreviations are marked with the full name at the first occurrence, and the abbreviations are used uniformly subsequently, in line with the writing norms of SCI papers.

Format Standardization: The hierarchical numbering, numerical range (en dash), unit format, and statistical terms all strictly follow the general requirements of international SCI journals, and the passive voice is uniformly used in the methodology section to meet the academic writing norms.

Ethical Normative Expression: The statements related to animal ethics, 3R principles, ARRIVE

guidelines, and AVMA euthanasia norms strictly follow the requirements of the International Committee of Medical Journal Editors (ICMJE), with no compliance risks.

Accuracy Guarantee: The official English names of institutions, companies, and guidelines, as well as the license numbers and ethics approval numbers, are accurately translated without any modification or omission, and the operation process is fully consistent with the original text.

Basic Information of Datasets

The basic information, acquisition platform and download time of the two included HCC public cohorts are shown in the table below:

**Dataset ID**	**Final Included Sample Size**	**Official Acquisition Platform**	**Core Data Type**
TCGA-LIHC	371 HCC cases	NCI Genomic Data Commons (GDC) Data Portal (https://portal.gdc.cancer.gov/)	Raw gene expression counts (HTSeq-Counts), complete clinical follow-up data, pathological characteristics, overall survival information
GSE76427	167 HCC cases	NCBI Gene Expression Omnibus (GEO) Database (https://www.ncbi.nlm.nih.gov/geo/)	Normalized gene expression matrix (GPL10558 platform, Illumina HumanHT-12 V4.0 expression beadchip), paired clinical and prognostic information

All data preprocessing steps were completed using R software (v4.3.2), and the standardized workflow was as follows:

Gene ID Conversion and Annotation: For the TCGA-LIHC dataset, Ensembl gene IDs were converted to official HGNC-approved gene symbols using the GENCODE v36 human genome annotation file; for the GSE76427 dataset, probe IDs were converted to gene symbols based on the GPL10558 platform annotation file, and the average expression value was taken for multiple probes mapped to the same gene.

Low-Expression Gene Filtering: Genes with an expression value of 0 in more than 50% of the total samples, as well as non-protein-coding genes, were excluded to reduce background noise interference.

Data Normalization: The raw count data of the TCGA-LIHC dataset was normalized by the Trimmed Mean of M-values (TMM) method via the edgeR R package (v3.42.4), and converted to

log2(CPM+1) format; the GSE76427 dataset was subjected to log2 transformation based on the original normalized matrix to ensure consistent data distribution between the two cohorts.

Batch Effect Correction: The ComBat algorithm in the sva R package (v3.50.0) was used to eliminate the cross-platform batch effect between the two datasets. The effectiveness of batch correction was verified by Principal Component Analysis (PCA), as shown in Figure 1H of the main text.

Integrated Matrix Construction: The common protein-coding genes in the two preprocessed datasets were extracted to construct the integrated gene expression matrix for subsequent differential expression analysis, correlation analysis and prognostic model construction.

Single-Cell Analysis

1. Integration and Preprocessing of scRNA-seq Data

In this study, single-cell RNA sequencing (scRNA-seq) datasets from 3 hepatocellular carcinoma (HCC) tissues (GSE107747, GSE223204) and 3 normal liver tissues (GSE223204, GSE174748) were collected and integrated from public databases. Quality control was performed using the Seurat package in R language. After filtering out low-quality cells, batch effects were removed and data integration was completed via the Harmony algorithm. Uniform Manifold Approximation and Projection (UMAP) algorithm was used for dimensionality reduction analysis. Based on the expression of classical marker genes, cells were precisely annotated into subpopulations including epithelial cells (cancer cells), immune cells (T cells, B cells, monocytes, etc.), and stromal cells (fibroblasts, endothelial cells, etc.).

2. Definition of FKBP9-related Cancer Cell Subgroups

To explore the effect of differential FKBP9 expression on the malignant biological behaviors of HCC and the tumor microenvironment (TME), we focused on the malignant epithelial cell (HCC cell) subpopulation. Based on the normalized expression level of FKBP9 gene in each single cell, cancer cells were divided into FKBP9-High and FKBP9-Low subgroups using the median-based method for subsequent comparative analysis and cell-cell interaction simulation.

3. Inference of Cell-cell Communication Network

The CellChat (v1.6.1) package was used to infer and systematically analyze the communication interactions between FKBP9-High/Low HCC cells and other cellular components in the TME (such as fibroblasts, monocytes, and T cells). The analysis was based on the CellChatDB.human database, covering information including secretory pathways, plasma membrane-bound ligands/receptors, and extracellular matrix (ECM) interactions.

4. Calculation of Ligand-Receptor Interaction and Role Analysis

By calculating the communication probability of each cell pair in specific signaling pathways, differential signaling patterns regulated by FKBP9 expression were identified. The statistical significance of ligand-receptor interactions was evaluated using permutation test. Network analysis tools were used to assess the roles of each cell population in specific pathways (such as TGF-β and MIF pathways), including the importance scores of sender, receiver, mediator, and influencer.

5. Data Visualization

Bubble plot was used to display significant ligand-receptor interaction pairs and their communication intensity. Chord diagram and hierarchy plot were used to visualize the spatial flow direction and interaction topology of specific pathways among different subgroups.

Statistical analysis

Data were presented as mean±standard deviation (SD) of at least three independent biological replicates. SPSS Statistics 23 and GraphPad Prism 5.0 software were used for statistical analysis and chart generation. Differences between two independent groups were assessed using Student's t-test when normally distributed (determined by D'Agostino-Pearson tests), including Welch correction in case of unequal variances (ascertained using F-test). If not normal, a nonparametric test (Mann–Whitney test) was used. One-way ANOVA followed by Tukey's post-hoc test was used for comparisons of multiple groups at a single time point. Two-way ANOVA followed by Tukey's post-hoc test was applied to repeated measures data with multiple time points, including cell proliferation curves, wound healing assays, tumor growth curves and protein half-life assays.

The survival curves were plotted using the Kaplan-Meier method, and the Log-rank test was used to compare survival differences between groups; univariate and multivariate Cox proportional hazards regression models were used to analyze independent factors influencing OS and DFS in HCC patients. Variables with $P < 0.05$ in the univariate Cox regression were included in the multivariate analysis. Prior to inclusion, multicollinearity among the independent variables was assessed using the variance inflation factor (VIF) and tolerance. The VIF $\geqslant 5$ was defined as indicative of multicollinearity, and variables exhibiting multicollinearity were selectively excluded. The P value < 0.05 was considered statistically significant.

Tables

Table S1 shRNAs Sequences

shRNA ID	Core target sequence (5'-3')	Target gene	GC
sh-NC	TTCTCCGAACGTGTCACGT	Scramble (no human gene target)	52.6%
sh-FKBP9-1	GCATTATTGGACCTCCATAAC	FKBP9 (NM_001145027)	42.9%
sh-FKBP9-2	GGACTTTCTCAGGTATCATTA	FKBP9 (NM_001145027)	38.1%
sh-FKBP9-3	GCACGTTTGACACGTACATTG	FKBP9 (NM_001145027)	47.6%
sh-c-MYC	GCTGGATTTCCTTTGGGCAAT	MYC (NM_001354870)	47.6%
sh-SEPT6	CCAGAAGCTGACCAATGAGTT	SEPT6 (NM_001012438)	47.6%
sh-GAPDH	GAAGGTGAAGGTCGGAGTCAA	GAPDH (NM_002046)	52.4%

Table S2 Primer Information

Genes	RT-PCR primers
h-FKBP9-F	GACGGCCAGAAGTTCGACTC
h-FKBP9-R	CGTTTACGCACATCCCAACAA
h-SEPT6-F	ATGGCAGCGACCGATATAGC
h-SEPT6-R	GGCTGACGGACTTATTCACCA
h-c-MYC-F	ATGGCCCATTACAAAGCCG
h-c-MYC-R	TTTCTGGAGTAGCAGCTCCTAA
h-YY1-F	CGCAGACGAAGGGTTTTGG
h-YY1-R	GAATTGATCCGAGGTTTTCTGGT
h-GAPDH-F	TGTGGGCATCAATGGATTTGG
h-GAPDH-R	ACACCATGTATTCCGGGTCAAT

Table S3 ChIP Primer Information

Genes	CHIP-primer	Target site location	c-MYC binding motif
SEPT6	c-MYC-SEPT6-chip-F1: CGCTGAGCAGAGTATGGGAGc-MYC-SEPT6-chip-R1: CCCGACCTCTGCTGAATTGT	-1911 bp ~ -1900 bp	ccccacatgcca
SEPT6	c-MYC-SEPT6-chip-F2: GGGTTGCAGGAAGAACTGGAc-MYC-SEPT6-chip-R2: GCACAGGAAACAGAGGAGCT	-1036 bp ~ -1029 bp	ccacctgc
SEPT6	c-MYC-SEPT6-chip-F3: GGAACCTGCCCTTTTGAGGAc-MYC-SEPT6-chip-R3: TCCCCTCTCTCTTGGGACTG	-38 bp ~ -27 bp	gcccacctgcgg

Table S4 Distribution of clinicopathological parameters in HCC patients (n=97)

Parameter	Result
Age (years)	56.2 ± 9.8
Sex	
Male	72 (74.2%)
Female	25 (25.8%)
Diameter (cm)	4.8 ± 2.3
MVI	
Present	36 (37.1%)
Absent	61 (62.9%)
PVT	
Present	19 (19.6%)
Absent	78 (80.4%)
Intrahepatic Metastasis	
Present	24 (24.7%)
Absent	73 (75.3%)
LNM	
Present	2 (2.1%)
Absent	95 (97.9%)
TNM	
Stage I	38 (39.2%)
Stage II	33 (34.0%)
Stage III	26 (26.8%)
Stage IV	0 (0%)
BCLC	
Stage 0	9 (9.3%)
Stage A	29 (29.9%)
Stage B	33 (34.0%)
Stage C	26 (26.8%)
Stage D	0 (0%)
Child	
Grade A	76 (78.4%)
Grade B	21 (21.6%)
Grade C	0 (0%)
Differentiation	
High	38 (39.2%)
Medium	42 (43.3%)
Low	17 (17.5%)
AFP (ng/mL)	31.2 (9.4-512.8)
CEA (ng/mL)	3.2 (1.5-8.7)
CA199 (U/mL)	18.5 (7.3-62.8)
HBV	
Positive	75 (77.3%)
Negative	22 (22.7%)

Table S5 Comparison of clinicopathological parameters between high and low FKBP9 expression groups

Parameter	Group	High FKBP9 (n=48)	Low FKBP9 (n=49)	χ^2	P
Age	≤60 years	29 (60.4%)	30 (61.2%)	0.012	0.913
	>60 years	19 (39.6%)	19 (38.8%)		
Sex	Male	35 (72.9%)	37 (75.5%)	0.118	0.731
	Female	13 (27.1%)	12 (24.5%)		
Diameter	≤5 cm	22 (45.8%)	31 (63.3%)	2.837	0.092
	>5 cm	26 (54.2%)	18 (36.7%)		
MVI	Present	22 (45.8%)	14 (28.6%)	4.102	0.043
	Absent	26 (54.2%)	35 (71.4%)		
PVT	Present	13 (27.1%)	6 (12.2%)	4.257	0.039
	Absent	35 (72.9%)	43 (87.8%)		
Intrahepatic Metastasis	Present	16 (33.3%)	8 (16.3%)	4.861	0.027
	Absent	32 (66.7%)	41 (83.7%)		
LNM	Present	2 (4.2%)	0 (0.0%)	2.085	0.149
	Absent	46 (95.8%)	49 (100.0%)		
TNM	I-II	29 (60.4%)	42 (85.7%)	7.840	0.005
	III-IV	19 (39.6%)	7 (14.3%)		
BCLC	0-A	13 (27.1%)	25 (51.0%)	5.742	0.049
	B-D	35 (72.9%)	24 (49.0%)		
Child	A	36 (75.0%)	40 (81.6%)	0.624	0.429
	B-C	12 (25.0%)	9 (18.4%)		
Differentiation	High-Med	38 (79.2%)	42 (85.7%)	0.897	0.343
	Low	10 (20.8%)	7 (14.3%)		
AFP	≤400 ng/mL	29 (60.4%)	38 (77.6%)	3.876	0.049
	>400 ng/mL	19 (39.6%)	11 (22.4%)		
CEA	≤5 ng/mL	37 (77.1%)	41 (83.7%)	0.821	0.365
	>5 ng/mL	11 (22.9%)	8 (16.3%)		
CA199	≤37 U/mL	35 (72.9%)	39 (79.6%)	0.893	0.345
	>37 U/mL	13 (27.1%)	10 (20.4%)		
HBV	Positive	37 (77.1%)	38 (77.6%)	0.003	0.955
	Negative	11 (22.9%)	11 (22.4%)		

Table S6 Comparison of clinicopathological parameters between high and low SEPT6 expression groups

Parameter	Group	High SEPT6 (n=47)	Low SEPT6 (n=50)	χ^2 /Fisher	P
Age	≤60 years	28 (59.6%)	31 (62.0%)	0.078	0.780
	>60 years	19 (40.4%)	19 (38.0%)		
Sex	Male	34 (72.3%)	38 (76.0%)	0.215	0.643
	Female	13 (27.7%)	12 (24.0%)		
Diameter	≤5 cm	21 (44.7%)	32 (64.0%)	3.982	0.046
	>5 cm	26 (55.3%)	18 (36.0%)		
MVI	Present	20 (42.6%)	16 (32.0%)	1.289	0.256
	Absent	27 (57.4%)	34 (68.0%)		
PVT	Present	12 (25.5%)	7 (14.0%)	2.371	0.123
	Absent	35 (74.5%)	43 (86.0%)		
Intrahepatic Metastasis	Present	15 (31.9%)	9 (18.0%)	3.154	0.076
	Absent	32 (68.1%)	41 (82.0%)		
LNM	Present	1 (2.1%)	1 (2.0%)	0.002	0.964
	Absent	46 (97.9%)	49 (98.0%)		
TNM	I-II	28 (59.6%)	43 (86.0%)	9.247	0.002
	III-IV	19 (40.4%)	7 (14.0%)		
BCLC	0-A	12 (25.5%)	26 (52.0%)	7.031	0.027
	B-D	35 (74.5%)	24 (48.0%)		
Child	A	35 (74.5%)	41 (82.0%)	0.836	0.361
	B-C	12 (25.5%)	9 (18.0%)		
Differentiation	High-Med	37 (78.7%)	43 (86.0%)	1.058	0.303
	Low	10 (21.3%)	7 (14.0%)		
AFP	≤400 ng/mL	28 (59.6%)	39 (78.0%)	4.573	0.032
	>400 ng/mL	19 (40.4%)	11 (22.0%)		
CEA	≤5 ng/mL	36 (76.6%)	42 (84.0%)	1.019	0.313
	>5 ng/mL	11 (23.4%)	8 (16.0%)		
CA199	≤37 U/mL	34 (72.3%)	40 (80.0%)	1.157	0.282
	>37 U/mL	13 (27.7%)	10 (20.0%)		
HBV	Positive	36 (76.6%)	39 (78.0%)	0.035	0.851
	Negative	11 (23.4%)	11 (22.0%)		

Table S7 Log-rank test and univariate Cox regression analysis for overall survival of HCC patients

Variable	Log-rank	Univariate Cox	
	P value	HR (95% CI)	P value
FKBP9	0.007	2.33 (1.22-4.33)	0.004
SEPT6	<0.001	2.12 (1.50-3.18)	<0.001
Age	0.301	1.11 (0.91-1.35)	0.211
Sex	0.401	1.79 (0.87-3.06)	0.342
Diameter	0.041	1.81 (1.13-2.45)	0.037
MVI	<0.001	2.64 (1.42-4.70)	0.002
PVT	0.312	1.42 (0.72-2.83)	0.320
Intrahepatic Metastasis	0.036	1.82 (1.18-2.76)	0.025
LNM	0.933	0.96 (0.34-2.73)	0.931
TNM	<0.001	3.81 (1.12-9.11)	0.003
BCLC	0.025	1.90 (1.10-3.42)	0.028
Child	0.402	1.33 (0.68-2.67)	0.411
Differentiation	0.001	2.81 (1.70-5.11)	<0.001
AFP	0.004	2.33 (1.13-4.42)	0.015
CEA	0.342	0.68 (0.33-1.25)	0.314
CA199	0.061	2.18 (0.82-4.10)	0.101
HBV	0.666	0.85 (0.41-1.84)	0.671

Table S8 Multicollinearity diagnosis of variables

Variable	Before removal		After removal	
	Variance Inflation Factor	Tolerance	Variance Inflation Factor	Tolerance
FKBP9 expression	5.632	0.178	3.247	0.308
SEPT6 expression	5.418	0.185	remove	
Tumor diameter	1.874	0.534	1.782	0.561
MVI	2.316	0.432	2.104	0.475
Intrahepatic metastasis	1.952	0.512	1.876	0.533
TNM stage	11.274	0.089	2.893	0.346
BCLC stage	10.892	0.092	remove	
Differentiation	1.743	0.574	1.652	0.605
Serum AFP level	1.685	0.593	1.594	0.627

Table S9 Multivariate Cox regression analysis for HCC patients

Variable	Overall Survival			Disease-Free Survival		
	HR	95% CI	P	HR	95% CI	P
**FKBP9**	2.13	1.24-4.12	0.011	2.95	1.72-5.42	0.004
**MVI**	1.84	0.86-2.95	0.082	1.97	1.08-2.91	0.047
**TNM**	4.14	2.43-6.03	<0.001	3.87	2.19-5.53	<0.001
**Differentiation**	1.65	0.91-2.75	0.469	1.77	0.85-3.33	0.638
**AFP**	1.33	0.94-2.68	0.362	2.41	0.83-3.59	0.317

Figures

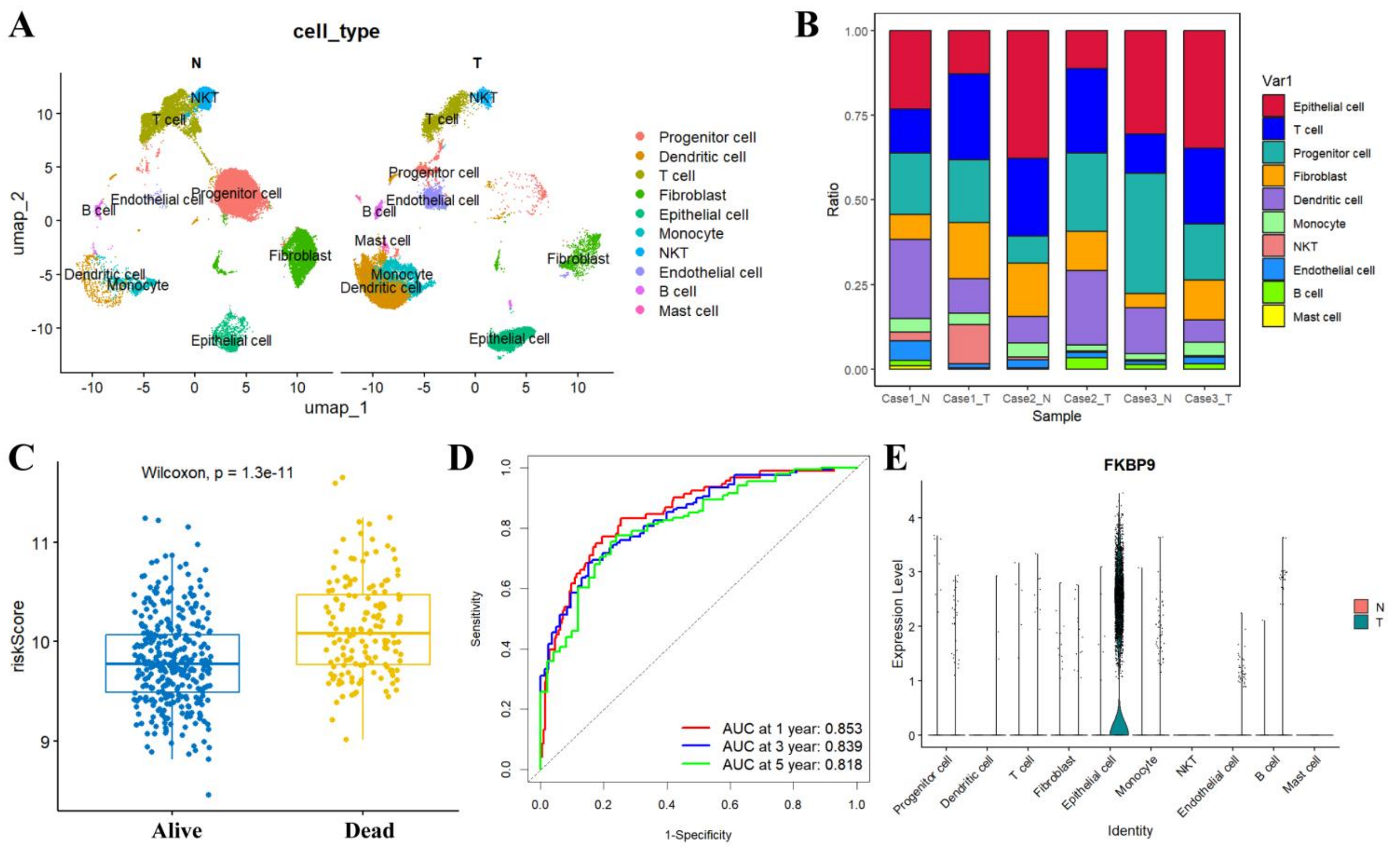

Figure S1 Quality control of single-cell RNA sequencing datasets, performance validation of the EMT-related prognostic model

(A) UMAP visualization of quality control metrics for integrated scRNA-seq datasets, including 3 HCC tumor tissues and 3 normal liver tissues; (B) Bar plot showing the relative proportion of each annotated major cell subpopulation across individual samples included in the integrated scRNA-seq cohort; (C-D) ROC curves evaluating the predictive performance of the 18-gene EMT-related prognostic model for 1-year, 3-year, and 5-year overall survival (OS) in HCC patients from the integrated TCGA-LIHC and GSE76427 bulk transcriptome cohort; (E) Violin plot showing the normalized expression level of FKBP9 across all annotated cell subpopulations in tumor and normal tissues of the integrated scRNA-seq dataset. Data are presented as mean ± SD; *p<0.05.**p < 0.01. ***p < 0.001. NS, no significance.

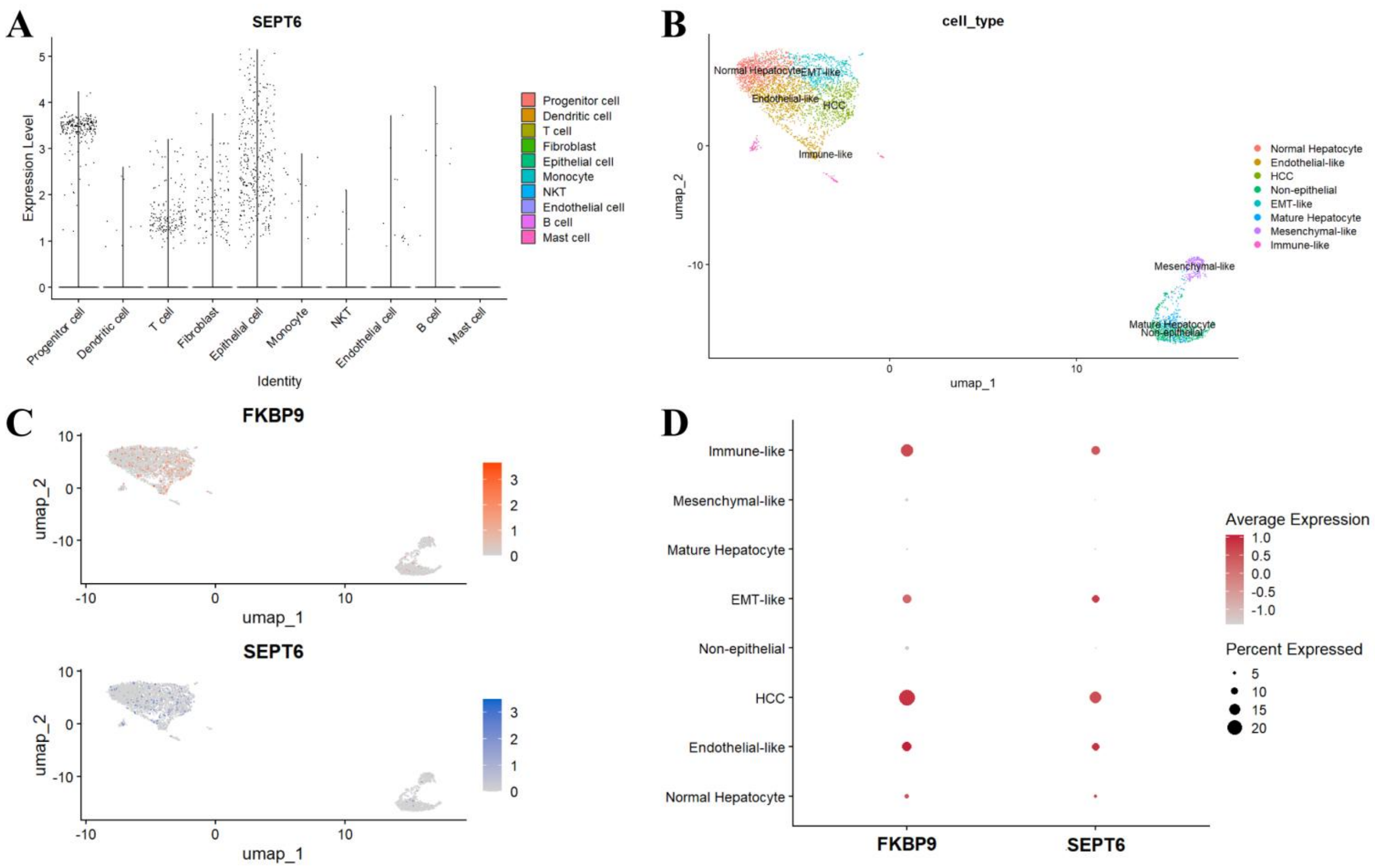

Figure S2 SEPT6 expression patterns at the single-cell level and its colocalization with FKBP9

(A) Violin plot showing the normalized expression level of SEPT6 across all annotated cell subpopulations in the integrated scRNA-seq dataset; (B-C) UMAP feature plots showing the co-expression pattern of FKBP9 and SEPT6 in the integrated scRNA-seq dataset.

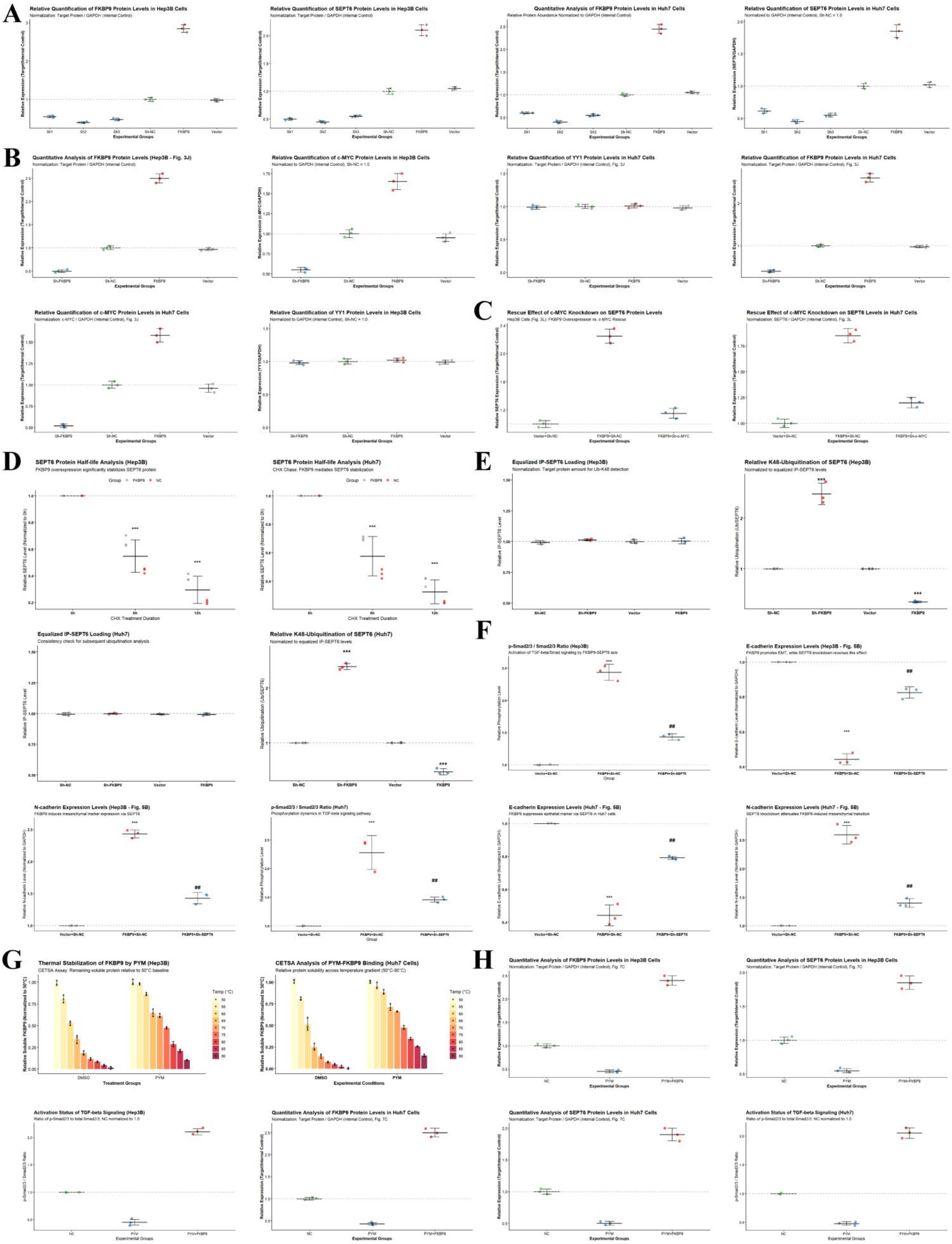

Figure S3 Quantitative Analysis of Western Blot Assays

(A) Quantification of changes in SEPT6 protein levels after FKBP9 knockdown detected by Western blot analysis; (B) Quantification of the effect of FKBP9 on the protein expression of

transcription factors c-MYC and YY1; (C) Quantification of the rescue experiment showing that c-MYC knockdown reverses the upregulation of SEPT6 protein induced by FKBP9 overexpression; (D) Quantification of the protein half-life assay based on cycloheximide (CHX) treatment to evaluate the degradation rate of SEPT6; (E) Quantification of the ubiquitination assay showing the effect of FKBP9 knockdown or overexpression on the level of K48-linked ubiquitination of SEPT6; (F) Quantification of the regulatory effect of the FKBP9-SEPT6 axis on the expression of phosphorylated Smad2/3 (p-Smad2/3) downstream of the canonical TGF-β pathway; (G-H) Quantification of thermal stability from the cellular thermal shift assay (CETSA) verifying the binding between Pinyonomycin and FKBP9. Data are presented as mean ± SD, n=3; *p<0.05.**p < 0.01. ***p < 0.001. NS, no significance.

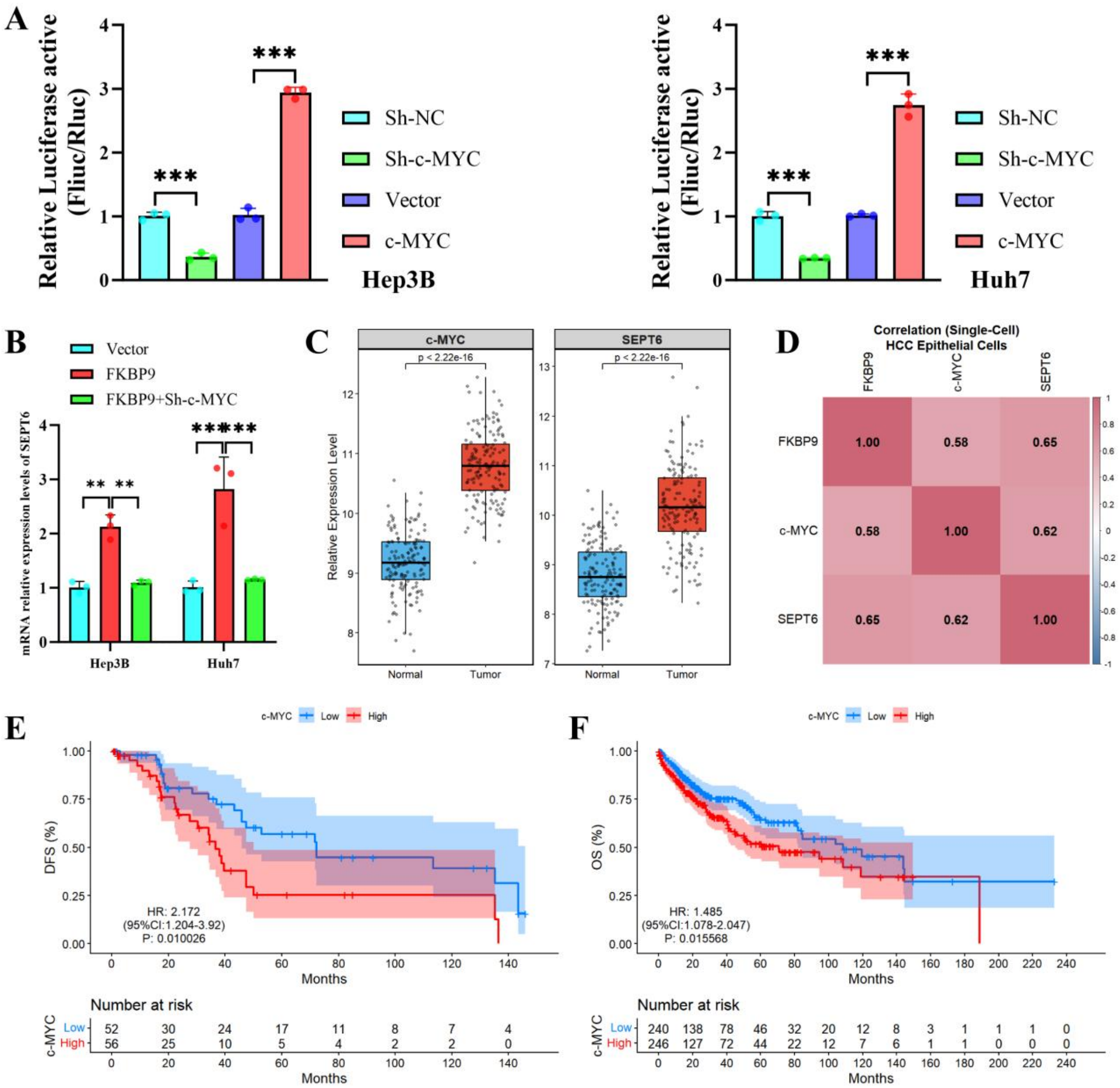

Figure S4 Correlation and Prognostic Analysis of the FKBP9/c-MYC/SEPT6 Axis in Hepatocellular Carcinoma

(A) A dual-luciferase assay demonstrated that c-MYC regulates the transcriptional activity of

SEPT6; (B) Quantitative real-time PCR (qPCR) results showing that c-MYC knockdown significantly reverses the upregulation of SEPT6 mRNA level induced by FKBP9 overexpression; (C) Validation of the universally upregulated expression trend of c-MYC and SEPT6 in HCC tissues based on The Cancer Genome Atlas and Gene Expression Omnibus databases; (D) Correlation heatmap showing the positive correlations among FKBP9, c-MYC and SEPT6 in the epithelial cell subpopulation derived from single-cell transcriptome data; (E-F) Kaplan-Meier survival curves showing the significant correlation between SEPT6 expression level and disease-free survival as well as overall survival in HCC patients. Data are presented as mean ± SD, n=3; *p<0.05.**p < 0.01. ***p < 0.001. NS, no significance.

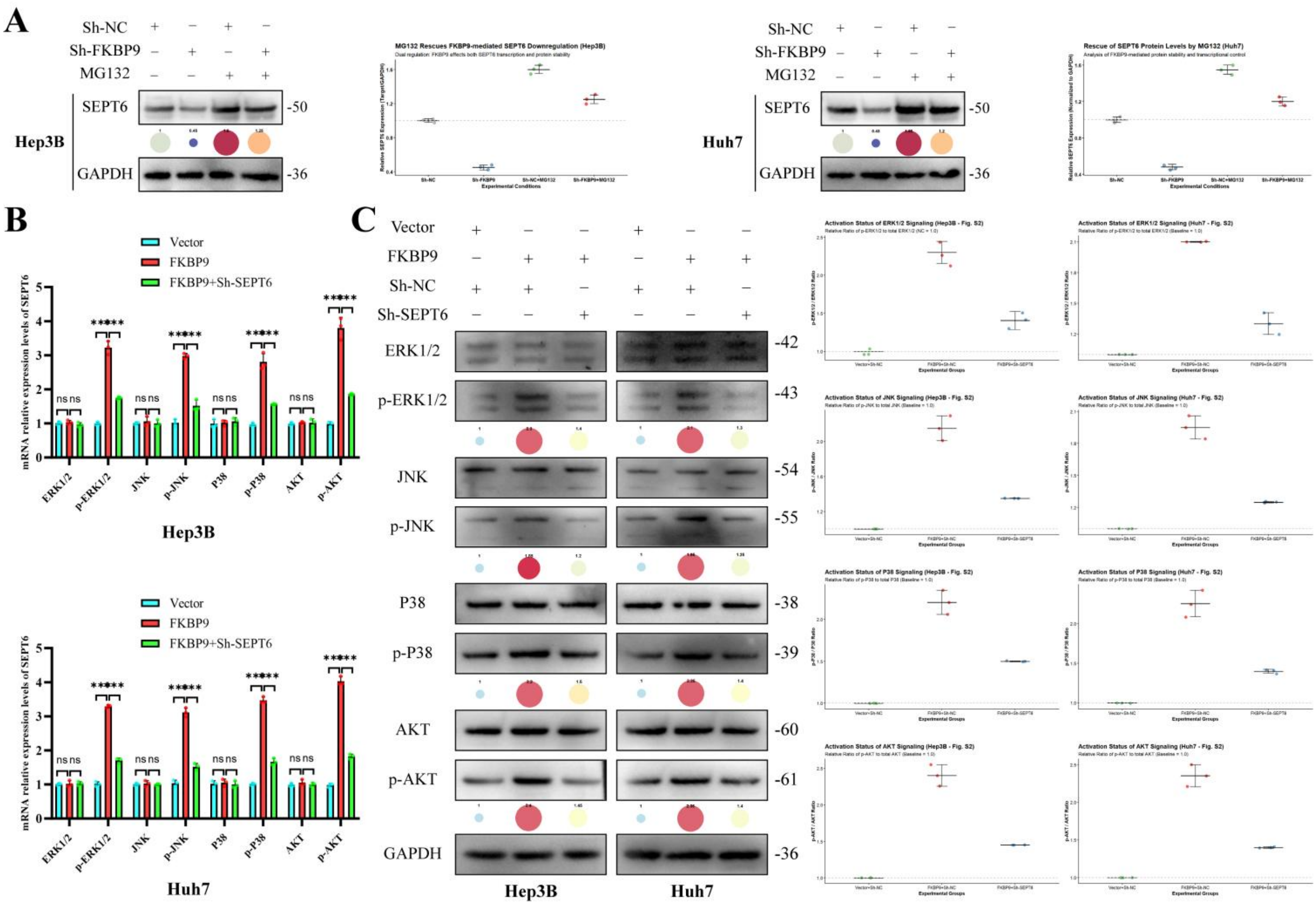

Figure S5 Regulation of FKBP9 on Protein Degradation and Non-canonical TGF-β Signaling Pathway

(A) Western blot results showing that treatment with the proteasome inhibitor MG132 significantly restores the reduced SEPT6 protein level induced by FKBP9 deficiency; (B-C) PCR and WB validation of the activating effect of the FKBP9-SEPT6 axis on TGF-β-induced non-canonical signaling pathways (including p-ERK1/2, p-JNK, p-p38 and p-Akt). Data are presented as mean ± SD, n=3; *p<0.05.**p < 0.01. ***p < 0.001. NS, no significance.

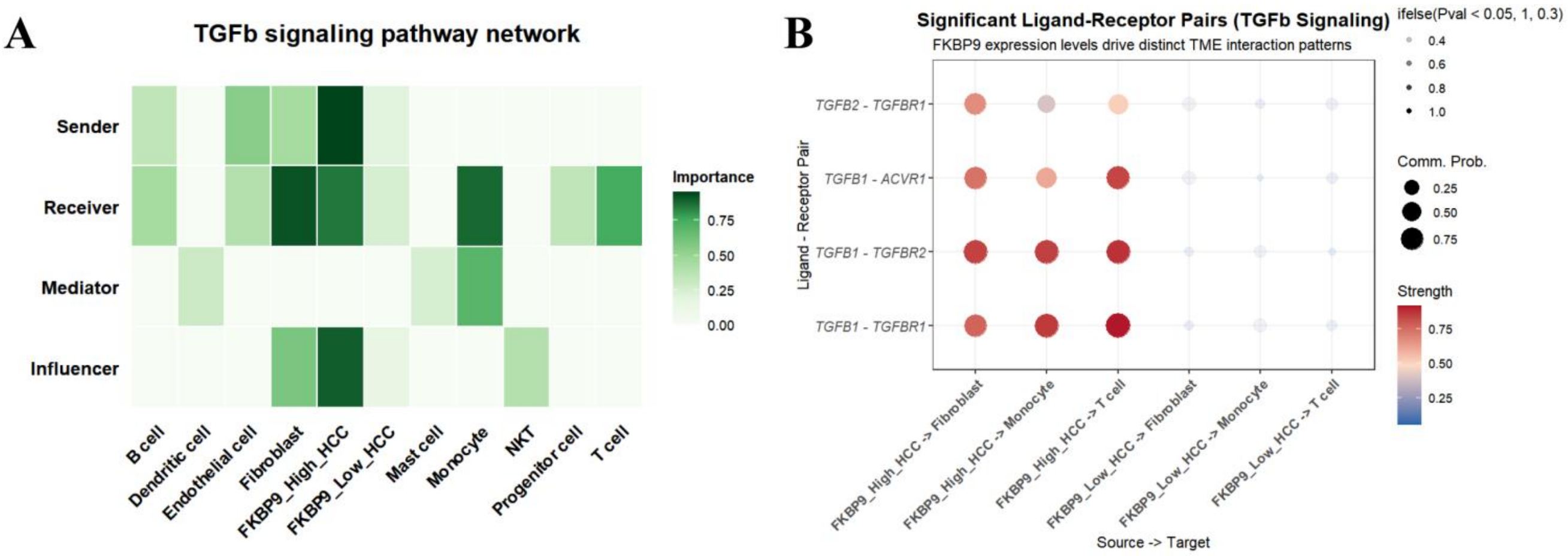

Figure S6 Analysis of Cell-Cell Communication and Paracrine Signaling in the HCC Microenvironment

(A) Bubble plot showing the cell-cell communication analysis at the single-cell level, which reveals the strong interactions between the FKBP9-high HCC subpopulation and fibroblasts as well as monocytes via paracrine TGF-β signaling; (B) Ligand-receptor pair interaction analysis showing the molecular basis of TGFB1 and its receptors (ACVR1/TGFBR1/TGFBR2) in driving extracellular matrix (ECM) remodeling and immunosuppression.

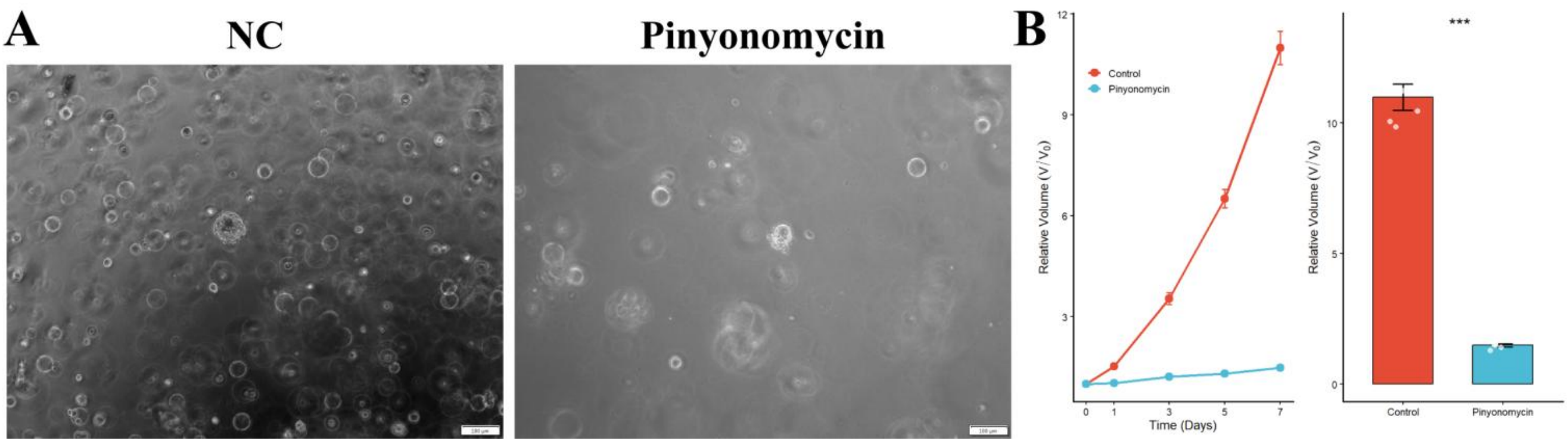

Figure S7 Pinyonomycin Inhibits the Growth of Hepatocellular Carcinoma Patient-derived Organoids

(A) Growth curves of HCC-PDOs treated with vehicle (Control) or 1 μg/mL Pinyonomycin. Relative volume (V/V_0) was calculated as the ratio of the current organoid volume to the initial volume on day 0, derived from the projected area measurements; (B) Quantification of relative volume of HCC-PDOs on day 7. Control HCC-PDOs exhibited approximately 2.1-fold increase in diameter, corresponding to an ~11-fold increase in volume over 7 days. In contrast, Pinyonomycin treatment significantly blocked organoid growth, with some organoids even showing shrinkage. Data are presented as mean ± SD, n=3; ***p < 0.001 versus the control group.
